# End User and Implementer Experiences of mHealth Technologies for Noncommunicable Chronic Disease Management in Young Adults: Systematic Review

**DOI:** 10.2196/jmir.8888

**Published:** 2017-12-12

**Authors:** Helen Slater, Jared M Campbell, Jennifer N Stinson, Megan M Burley, Andrew M Briggs

**Affiliations:** ^1^ School of Physiotherapy and Exercise Science Curtin University Perth Australia; ^2^ Joanna Briggs Institute Faculty of Health Science University of Adelaide Adelaide Australia; ^3^ Lawrence S Bloomberg Faculty of Nursing University of Toronto Toronto, ON Canada; ^4^ The Hospital for Sick Children Toronto, ON Canada; ^5^ Health Networks Department of Health Government of Western Australia Perth Australia

**Keywords:** musculoskeletal pain, health services research, telemedicine, noncommunicable disease, chronic disease, health policy

## Abstract

**Background:**

Chronic noncommunicable diseases (NCDs) such as asthma, diabetes, cancer, and persistent musculoskeletal pain impose an escalating and unsustainable burden on young people, their families, and society. Exploring how mobile health (mHealth) technologies can support management for young people with NCDs is imperative.

**Objective:**

The aim of this study was to identify, appraise, and synthesize available qualitative evidence on users’ experiences of mHealth technologies for NCD management in young people. We explored the perspectives of both end users (young people) and implementers (health policy makers, clinicians, and researchers).

**Methods:**

A systematic review and meta-synthesis of qualitative studies. Eligibility criteria included full reports published in peer-reviewed journals from January 2007 to December 2016, searched across databases including EMBASE, MEDLINE (PubMed), Scopus, and PsycINFO. All qualitative studies that evaluated the use of mHealth technologies to support young people (in the age range of 15-24 years) in managing their chronic NCDs were considered. Two independent reviewers identified eligible reports and conducted critical appraisal (based on the Joanna Briggs Institute Qualitative Assessment and Review Instrument: JBI-QARI). Three reviewers independently, then collaboratively, synthesized and interpreted data through an inductive and iterative process to derive emergent themes across the included data. External validity checking was undertaken by an expert clinical researcher and for relevant content, a health policy expert. Themes were subsequently subjected to a meta-synthesis, with findings compared and contrasted between user groups and policy and practice recommendations derived.

**Results:**

Twelve studies met our inclusion criteria. Among studies of end users (N=7), mHealth technologies supported the management of young people with diabetes, cancer, and asthma. Implementer studies (N=5) covered the management of cognitive and communicative disabilities, asthma, chronic self-harm, and attention deficit hyperactivity disorder. Quality ratings were higher for implementer compared with end user studies. Both complementary and unique user themes emerged. Themes derived for end users of mHealth included (1) Experiences of functionality that supported self-management, (2) Acceptance (technical usability and feasibility), (3) Importance of codesign, and (4) Perceptions of benefit (self-efficacy and empowerment). For implementers, derived themes included (1) Characteristics that supported self-management (functional, technical, and behavior change); (2) Implementation challenges (systems level, service delivery level, and clinical level); (3) Adoption considerations for specific populations (training end users; specific design requirements); and (4) Codesign and tailoring to facilitate uptake and person-centered care.

**Conclusions:**

Synthesizing available data revealed both complementary and unique user perspectives on enablers and barriers to designing, developing, and implementing mHealth technologies to support young people’s management of their chronic NCDs.

**Trial Registration:**

PROSPERO CRD42017056317; http://www.crd.york.ac.uk/PROSPERO/display_record.asp?ID=CRD 42017056317 (Archived by WebCite at http://www.webcitation.org/6vZ5UkKLp)

## Introduction

Young people are digital natives, and the portability and capabilities of digital technologies can act as a lever to connect them to health systems. This capability to connect is especially important for young people with chronic noncommunicable diseases (NCDs) during the critical transition from childhood to young adulthood [1,2].

### Young People’s Use of Mobile Technologies to Support Self-Management of Chronic NCDs

We have previously identified how mobile health (mHealth) technologies could support self-management of young people with persistent musculoskeletal pain who are making this transition [2,3] and how to specifically address their self-management needs by improving access to disease information, strategies to manage symptoms, and social support [4]. Self-management is well recognized as a fundamental component of chronic NCD care, denoting the active participation of people in their care with the aim of minimizing the impact of chronic disease on physical health status and functioning and enabling people to cope with the psychological effects of illness [5]. Core self-management skills include problem solving, decision making, resource utilization, forming patient-health professional relationships, taking action, and self-tailoring, all skills that can be feasibly supported by appropriate mHealth technologies as highlighted in findings from a recent systematic review on this issue [1]. Furthermore, the use of mHealth technologies as an enabler to self-management is an intuitive choice for young people, given the high rates of Internet usage globally, with rates nearing 100% for the millennial generation in many of the world’s largest economies [6]. Young people are also more likely than older generations to own a mobile phone in virtually every country [6]. Digital technologies can also provide a potential mechanism to help mitigate care disparity [7], reaching across high, middle, and low-income economies [8] to enable the delivery of integrated, holistic information about chronic NCD management [9].

### Evidence-Practice and Policy-Practice Gaps for the Use of Mobile Health Technologies to Support Self-Management of Chronic NCDs

Although the use of mHealth technologies, including mobile apps, to support self-management of NCDs has also grown substantially [10], the evaluation of their quality, safety, and outcomes indicate that significant evidence-practice and policy-practice gaps remain [1,11,12]. In particular, there is a dearth of high-quality evidence on the use of mHealth technologies to support young people’s self-management of their persistent musculoskeletal pain conditions [2,13]. Recent efforts address some of these gaps, providing evidence for how mHealth apps can improve the access of young people with chronic pain to disease information, facilitate symptom management and social support [4], and support their self-management of cancer pain [14,15]. In the context of young people’s use of mHealth to support their management of other chronic NCDs (asthma, diabetes, and cancer), findings from a recent systematic review indicate the need for more high-quality studies targeting the development, evaluation, use, and effectiveness of mobile apps [1]. One significant issue common to mHealth interventions is that they fail to be fully embedded into real-world settings and scaled up, with many studies being conducted as pilots or feasibility trials [1,16]. Another key finding from this same review emphasized the critical role of codesign of mobile apps. This means bringing together both end users (here, young people) and implementers (policy makers or health professionals tasked with implementation) to ensure meaningful design and to facilitate strong engagement, adoption, and sustained uptake [17]. Codesign includes consideration of factors such as feasibility, engagement, ease of use, ease of navigation, ease of understanding, satisfaction, acceptability, reliability, functionality, aesthetics, information quality, and subjective quality [1,14,15,18,19].

### Why This Study?

The primary motivation for this systematic review was to inform appropriate mHealth resource design, evaluation, and implementation specifically targeted for young people with chronic NCDs including persistent musculoskeletal pain. The experiences of young people with chronic NCDs diseases were considered more broadly, as the self-management of chronic conditions frequently overlaps and is associated with comorbidities and multi-morbidities [20,21] requiring similar core self-management skills [5]. To optimally inform implementation approaches, a comprehensive understanding of users’ experiences and perceptions is essential. Qualitative (including mixed methods) studies are likely to provide the richest insights, and such perspectives and insights are recognized as a critical component of implementation approaches related to interventions and system-wide models of care [22,23]. Additionally, as the implementation of new interventions is recommended to be a partnered process between end users and implementers, identifying unique and overlapping user perspectives could lead to better shared decision making and care integration [18].

This systematic review therefore had two key aims: (1) to identify users’ (end user and implementers) experiences with mHealth technologies to support the self-management of young people with chronic NCDs, and (2) to identify what factors these users (end user and implementers) perceived or experienced as facilitators or barriers to the uptake and implementation of mHealth technologies for young people with chronic NCDs.

## Methods

### Conduct of Systematic Review

This systematic review followed an a priori published protocol with detailed methods [13]. Our review is reported in accordance the Preferred Reporting Items for Systematic Review and Meta-Analysis (PRISMA) statement checklist [24] and Enhancing Transparency in Reporting the Synthesis of Qualitative Research (ENTREQ) checklist [25] (Multimedia Appendices 1 and 2). This systematic review followed an a priori published protocol with detailed methods [13] and can be found at: http://www.crd.york.ac.uk/PROSPERO/ display_record.asp?ID=CRD42017056317.

### Eligibility Criteria

#### Types of Participants

This review considered all qualitative studies on young people (in the age range of 15-24 years) with chronic NCDs (end users), which included technologies intended for use by patients [13]. Studies were included where ≥50% of the cohort met the age criteria or where the mean age range (rounded) of participants fell within the 15 to 24 year age range. Additionally, the experiences and perspectives of “Implementers” (defined as including health service delivery providers, administrators, researchers, clinicians, and policy makers) supporting young people with chronic NCDs were included and considered separately.

Chronic NCDs were defined as conditions of long duration and generally slow progression, lasting 3 months or more and included, but were not limited to, musculoskeletal conditions, diabetes, respiratory conditions (such as asthma), cardiovascular diseases, mental health disorders, and cancer [26].

#### Phenomena of Interest

This review considered studies that evaluated the use of mHealth technologies to support young people manage their chronic NCDs [13]. To be included, studies needed to have evaluated users’ (implementers and end users) (1) perspectives or experiences (ie, perceptions of feasibility, engagement, ease of use, ease of navigation, ease of understanding, satisfaction, acceptability, reliability, functionality, aesthetics, information quality, and subjective quality) of using mHealth technologies to support the management of chronic NCDs and (2) factors that users (end user and implementers) perceived or experienced as facilitators or barriers to the uptake and/or implementation of mHealth technologies for young people with chronic NCDs [13]. In this review, mHealth included any mobile device or service, such as mobile phones, short message service (SMS), smartphones, personal digital assistants, and devices that work on wireless technology or Bluetooth-compatible devices [27]. Interventions delivered using a Web-based platform were included only if it was specified that the patient accessed the service via a mobile phone or other mobile device.

#### Context

Studies carried out in any setting were considered. The rationale included the portable and accessible nature of mHealth technologies, which enables varied use not just within different care settings by different patients but extending across different contexts by the same patient (ie, continuing to access and utilize the same mobile phone app in the community [locally and remotely] in primary care and tertiary care settings).

#### Types of Studies

This review considered primary research studies that used qualitative methods to collect and analyze data, including but not limited to phenomenology, grounded theory, ethnography, critical enquiry, participatory action research, and descriptive qualitative studies. The qualitative components of mixed-methods studies were also included.

### Search Strategy

A three-step search strategy was utilized in this review [13]. An initial limited search of MEDLINE (PubMed) and CINAHL and PsycINFO was to be undertaken, followed by analysis of the text words contained in the title and abstract and the index terms used to describe an article. A second search using all identified keywords and index terms was then undertaken across all databases including EMBASE, MEDLINE (PubMed), Scopus, and PsycINFO. Two independent academic research librarians were consulted to provide feedback on the final search strategy. The search for gray literature included ProQuest Dissertations and Theses, KT, Epistemonikos, as well as health policy and nongovernmental organization literature based on the research team’s knowledge. Third, the reference list of all included reports and articles were hand searched for additional studies. Studies published in English were considered for inclusion in this review. The search was carried out in December 2016 by a senior review methodologist (JC). Studies from 2007 were included to align with global access to 147 Wideband Code-Division Multiple Access; the standard found in third generation mobile telecommunications and available globally [28].

Initial keywords used were chronic, long term, persistent, noncommunicable, disease, respiratory, asthma, cystic fibrosis, lung disease, diabetes, cancer, heart disease, cardiovascular disease, pain, muscular disease, joint diseases, musculoskeletal, kidney disease, young, adolescent, adolescence, eHealth, mHealth, mobile application, mobile health app, mobile health application, smartphone application, digital technologies, intervention, qualitative, experience, phenomenology, grounded theory, action research, implementation, implementer, and end user. The full search strategies are included in Multimedia Appendix 3.

### Screening and Selection

#### Overview

Search results were collated in a reference database (Endnote X7 version 3.1, Thomson Reuters, New York), duplicates were deleted, and initial screening of titles and abstracts was conducted by one reviewer (JC), followed by the retrieval of full texts. Full texts were then reviewed against the inclusion criteria by two independent reviewers (HS and JC) to confirm eligibility. Disagreements were resolved through discussion.

#### Assessment of Methodological Quality

Papers selected for retrieval were assessed by two independent reviewers (JC and HS) for methodological quality before inclusion using the standardized critical appraisal instrument for qualitative research from the Joanna Briggs Institute, JBI-QARI [29]. Studies were not excluded on the basis of quality ratings. Any disagreements were resolved through discussion until consensus was reached.

#### Data Extraction

Data were extracted by one reviewer (JC) from papers included in the review using the standardized extraction tool from JBI-QARI [29]. A second reviewer (HS) also completed data extraction for 30% of articles to confirm congruence. The primary focus of data extraction was the identification of specific qualitative findings—reported themes, subthemes, and metaphors—related to the phenomena of interest, which were subsequently synthesized as described below. Additionally, descriptive data, including details about the mHealth apps, study methods, country of development, and age range of participants were extracted.

The credibility of findings was assessed based on how they were supported in the text [29], as follows:

- Unequivocal: findings accompanied by an illustration that is beyond reasonable doubt and therefore not open to challenge.
- Credible: findings accompanied by an illustration lacking clear association with it and therefore open to challenge.
- Unsupported: findings not supported by data.

#### Data Synthesis

A meta-synthesis approach was used to organize and interpret pooled data [29]. Initially, three reviewers (JC, AMB, and HS) familiarized themselves with the extracted data and independently developed preliminary categorizations. At a subsequent 3-day workshop, these independently and deductively derived categories were presented, discussed, and iteratively and inductively organized into consensus-based descriptive themes from which we derived new, higher-order themes that extended beyond the findings of primary studies. Findings were linked back to the research questions to ensure relevance and appropriate contextualization. Themes were then subjected to a meta-synthesis to inform declarative statements that could be applied as an evidence-base to our research aims. Four members of the team (AMB, JC, MB, and HS) participated in the meta-synthesis. Findings based on the experiences of end users and implementers were meta-synthesized separately and compared and contrasted.

On the basis of consensus, a reporting framework was developed to reflect these synthesized findings. The reporting framework was populated with derived themes and supporting evidence from primary study findings. To ensure external validity, one member of the team (JS) with substantial clinical and research expertise in the development and implementation of digital technologies for young people with chronic conditions provided independent feedback over the meta-synthesis process. Where relevant, findings and supporting evidence were adjusted to reflect a consensus decision, and the reporting framework was refined. Finally, a systems and health policy expert (MB) was engaged to assist with final policy and practice recommendations, with a final round of independent review (JS) conducted as outlined previously.

## Results

### Identification and Selection

**Figure 1:**
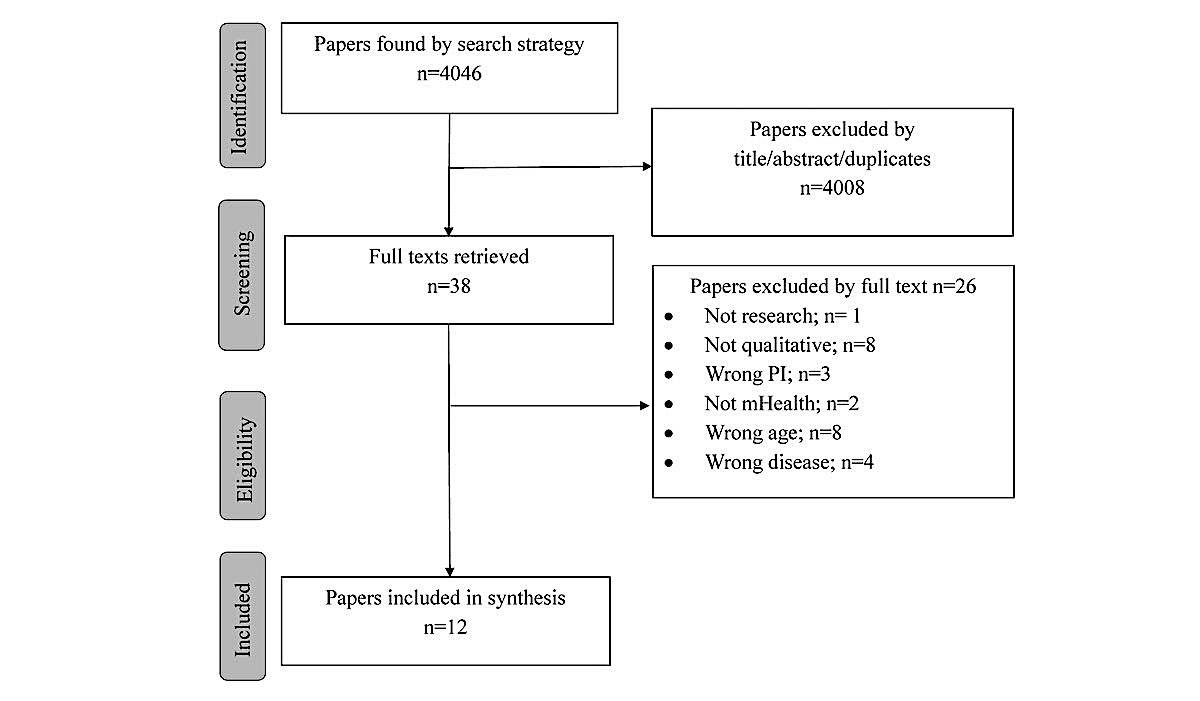
Flow diagram of study identification and selection adapted from preferred reporting items for systematic review and meta-analysis (PRISMA) flowchart. PI=phenomenon of interest; mHealth=mobile health.

The initial search identified 4046 potential studies from which 1193 studies were excluded as duplicates and 2815 were excluded based on the review on their titles or abstracts (Figure 1).

Overall, 38 studies were identified as potentially meeting the inclusion criteria based on the review of their titles and abstracts. From these, 12 studies were ultimately included [30-41]. Reasons for exclusion included not being a research paper [42], not being qualitative or having a qualitative component [43-50], investigating the wrong phenomena of interest [51-53], not meeting the definition of mHealth [54,55], the population being outside the target age band [19,56-62], and the population being affected by a condition not considered to be a chronic NCD (eg, mHealth promotion interventions with no specific chronic NCD or lifestyle behaviors) [63-66]. Seven studies contributed findings on end users [30,31,33-35,37,39], whereas 5 studies [32,36,38,40,41] reported on implementers.

### Included Study Characteristics

Characteristics of included studies are described in Table 1 (end user studies) and Table 2 (implementer studies). Among end users, mHealth technologies were applied to aid in managing diabetes [30,34,35], cancer (chemotherapy symptom management) [31,37], and asthma [33,39]. Implementers included occupational therapists [32], speech language pathologists [32], nurses [36], physicians [36,40], as well as medical [38,41] and nonmedical [38,41] health care professionals assisting in the management of cognitive and communicative disabilities [32], asthma [36,40], chronic self-harm [38], and attention deficit hyperactivity disorder (ADHD) [41]. Studies on end users were carried out in the United Kingdom [30,37], United States [31,33,39], and Norway [34,35], whereas studies on implementers were conducted in the United Kingdom [38,41], United States [36,40], and Sweden [32].

**Table 1:** Characteristics of included end user studies.

End user studies	Phenomena of interest	Participants	mHealth^a^ technology used	Method or design^b^; AA^c^	Setting; Geographic location
Ashurst et al 2014 [30]	Use of an app to help prepare for clinical appointments	Young people with type 1 diabetes; aged 16 to 25 years; mean age 20.3 years	Apps developed by young people with diabetes to facilitate agenda setting in clinic consultations, data logging and insulin dose calculation	Open-ended questions (email and web-based); AA: Inductive conventional content analysis; summative content analysis	Community; United Kingdom
Baggott et al 2012 [31]	Perceptions about using mobile oncology symptom tracker (mOST) and any technical difficulties they experienced	Adolescents and young adults with cancer; 13 to 21 years; receiving chemotherapy; mean age 18.2 years	A mobile phone–based electronic symptom diary (mOST)	Interviews and questionnaire; AA: Not specified	Pediatric hospitals; inpatient or clinic settings; United States
Carpenter et al 2016 [33]	How app features promote self-observation, self-judgment and foster positive self-reflection; app features work synchronously to increase adolescents’ asthma self-management and improve outcomes	Convenience sample of 20 adolescents with asthma; 12 to 17 years; mean 14.7 years; >50% over 15 years	Two asthma self-management apps (one targeted to adults and one to children)	20 to 30 min telephone interview with verbatim transcription; AA: Framework synthesis based on a framework analysis (self-regulation theory)	Pediatric practice located in an urban area; United States
Froisland and Arsand 2015 [34]	To evaluate the effect of the designed tool with regard to empowerment, self-efficacy, and self-treatment	Adolescents with type 1 diabetes; 13 to 19 years; mean age 16.2 years; >50% over 15 years	Mobile phone–based tool designed to capture and visualize adolescent food intake to affect understanding of calorie counting and help doctor-adolescent communication	Semistructured interview; AA: Deductive approach based on empowerment theory	Pediatric clinic; Norway
Froisland et al 2012 [35]	Adolescent patients’ experiences with two different mobile phone apps used for diabetes care	Adolescents with type 1 diabetes; 13 to 19 years; mean 16.2 years	App that contained a visual or picture-based diabetes diary to record physical activity, food eaten that communicated with glucometer and Web-based SMS^d^ used to contact providers and receive educational messages	Structured interview (transcribed) with field notes; AA: Inductive qualitative description influenced by phenomenology and hermeneutics	Pediatric clinics; Norway
Gibson et al 2010 [37]	Key benefits of the Advanced Symptom Management System (ASyMS-YG)	Young people; inpatient intravenous chemotherapy; 13 to 18 years; median age 15 years; >50% over 15 years)	ASyMS: through which patients can report chemotherapy-related symptoms through mobile	Questionnaires and semistructured interviews; AA: Thematic analysis	Cancer units; United Kingdom
Rhee et al 2014 [39]	Feasibility and user acceptability of mobile phone–based asthma self-management aid for adolescents (mASMAA)	Adolescents with asthmas; Adolescent-parent dyads; 13 to 17 years; mean 15.1 years; >50% over 15 years	mASMAA which facilitates symptom monitoring, treatment adherence, and adolescent patent partnership	Focus groups; semistructured questions (recorded and transcribed); AA: Content analysis	Clinical setting (emergency department and primary care clinics in a university medical center); United States

^a^mHealth: mobile health.

^b^Qualitative design or study type is specified where explicitly stated within studies, otherwise descriptive detail is provided.

^c^AA: analytic approach.

^d^SMS: short message service.

**Table 2:** Characteristics of included implementer studies.

Implementer studies	Phenomena of interest	Participants	mHealth^a^ technology used	Method or design^b^; AA^c^	Setting; Geographic location
Buchholz et al 2013 [32]	Professionals’ views of satisfaction, participation, and involvement in daily life of adolescents and adults with communicative disabilities who tried texting with picture symbols and speech synthesis through mobile phones	Four occupational therapists and three speech language pathologists who had worked with end users (adolescents and adults with cognitive and communicative disabilities using the intervention)	Texting with picture symbols and speech synthesis in mobile phones	Semistructured interview with independent transcription; AA: Retrospective qualitative analysis theory influenced by directed content analysis	Community setting; Sweden
Geryk et al 2016 [36]	The use of attitudes and preferences for asthma mHealth app features among parents and clinicians	20 caregivers and 6 clinicians involved in the care of adolescents with asthma	Two asthma self-management apps (one targeted at adults and one at children)	Questionnaires and interviews; AA: Thematic analysis	Pediatric practices; United States
Owens and Charles 2016 [38]	Barriers to recruitment and implementation of a texting intervention for adolescents who self-harm	Clinicians and service managers working in child and adolescent mental health services (CAHMS) with adolescents who self-harm	An SMS text messaging (short message service), (TeenTEXT) that delivered, scheduled, or prompted personalized messages	Field notes and focus groups; AA: Inductive thematic analysis	CAHMS; United Kingdom
Schneider et al 2014 [40]	Physicians’ views on patient-provider communication with their adolescent asthma patients, mechanisms for relating better with patients, their use of mobile technologies, and willingness to integrate technology in patient care	Residents and attending physicians about mHealth use for adolescents’ management of asthma	Mobile technology for patient care (no one specific tool or technology)	Interviews (with recording and transcription); AA: Constant comparative method using a priori codes	One pediatric group in an urban academic medical center; United States
Simons et al 2016 [41]	To explore patients’ and health care professionals’ views regarding the use of remote monitoring technology (RMT) during medication titration for attention deficit hyperactivity disorder (ADHD)	Health care professionals working with people with ADHD	RMT for people undergoing ADHD medication titration which sent automated text messages (linking to questionnaires)	Exploratory cross-sectional focus group; AA: Thematic analysis and charting were used to search for data patterns within and across participant groups	Four National Health Service mental health providers; United Kingdom

^a^mHealth: mobile health.

^b^Qualitative design or study type is specified where explicitly stated within studies, otherwise descriptive detail is provided.

^c^AA: analytic approach.

### Methodological Quality Assessment

Table 3 shows the findings of the critical appraisal for studies of end users (n=7) and implementers (n=5), respectively. Studies on implementers were scored as higher quality than those on end users.

This was particularly true for question 8 on the representation of participant voices, which were adequately represented for all 5 studies on implementers but only for 4 of the 7 studies on end users. Researchers’ cultural or theoretical backgrounds were inconsistently reported (question 6), whereas the impact of the researcher on the research was rarely addressed (question 7).

### Data Analysis and Meta-Synthesis

Results of the meta-synthesis are presented below. Data are presented as a synthesized finding with supporting themes and component subthemes (for a summary of themes or subthemes, see Table 4). Results are reported separately for end users and implementers. Examples of supporting evidence are provided in Textboxes along with statements about level of credibility. Data were subsequently examined for complementarity, indicating both common and unique user themes, which subsequently informed recommendations for policy and practice. Full supporting data and original findings are presented in Multimedia Appendices 4 and 5.

### End Users’ Experiences and Perspectives

#### Theme 1. Functionality of mHealth Technology

End users perceived the functionality of mHealth technologies as important; specifically, subthemes related to (1) functionality as an important enabler to supporting self-management and (2) person-centered clinical encounters (Textbox 1).

**Table 3:** Critical appraisal of the methodological quality of included studies.

Users		Question number									
		Q1^a^	Q2^b^	Q3^c^	Q4^d^	Q5^e^	Q6^f^	Q7^g^	Q8^h^	Q9^i^	Q10^j^
**End users**											
	Ashurst et al 2014 [30]	Y^k^	Y	Y	Y	Y	N^l^	N	N	Y	Y
	Baggott et al 2012 [31]	U^m^	U	U	N	U	N	N	N	Y	U
	Carpenter et al 2016 [33]	Y	Y	Y	Y	Y	N	N	Y	Y	Y
	Froisland and Arsand 2015 [34]	Y	Y	Y	Y	Y	Y	N	N	Y	Y
	Froisland et al 2012 [35]	Y	Y	Y	Y	Y	Y	N	Y	Y	Y
	Gibson et al 2010 [37]	Y	Y	Y	Y	N	Y	N	Y	Y	Y
	Rhee et al 2014 [39]	U	Y	Y	Y	Y	U	N	Y	Y	Y
	Positive/7	5	6	6	6	5	3	0	4	7	6
**Implementers**											
	Buchholz et al 2013 [32]	Y	Y	Y	Y	Y	N	N	Y	Y	Y
	Geryk et al 2016 [36]	Y	Y	Y	Y	Y	Y	N	Y	Y	Y
	Owens and Charles 2016 [38]	Y	Y	Y	Y	Y	Y	N	Y	Y	Y
	Schneider et al 2014 [40]	U	Y	Y	Y	Y	N	N	Y	Y	Y
	Simons et al 2016 [41]	Y	Y	Y	Y	Y	Y	Y	Y	Y	Y
	Positive/5	4	5	5	5	5	3	1	5	5	5

^a^Q1: Is there congruity between the stated philosophical perspective and the research methodology?

^b^Q2: Is there congruity between the research methodology and the research question or objectives?

^c^Q3: Is there congruity between the research methodology and the methods used to collect data?

^d^Q4: Is there congruity between the research methodology and the representation and analysis of data?

^e^Q5: Is there congruity between the research methodology and the interpretation of results?

^f^Q6: Is there a statement locating the researcher culturally or theoretically?

^g^Q7: Is the influence of the researcher on the research, and vice-versa, addressed?

^h^Q8: Are participants, and their voices, adequately represented?

^i^Q9: Is the research ethical according to current criteria or for recent studies, and is there evidence of ethical approval by an appropriate body?

^j^Q10: Do the conclusions drawn in the research report flow from the analysis, or interpretation, of the data? [29].

^k^Y=yes.

^l^N=no.

^m^U=unclear.

**Table 4:** Summary of themes and subthemes derived for end users and implementers.

User group	Themes	Subthemes
End users	Functionality of mHealth^a^ technology	mHealth functionality to support self-management
		mHealth functionality to support young person-centered clinical encounters
	Acceptance of mHealth technologies	Perceptions of technical usability
		Perceptions and experiences around acceptability and feasibility
	The importance of codesign	Intrapersonal factors
		Extra-personal factors
	Perceptions of benefit	Self-efficacy
		Empowerment
Implementers	mHealth characteristics that support young people’s management of noncommunicable diseases	Functional aspects of design that support end users’ management
		Technical characteristics can help their delivery of clinical care
		mHealth can support positive health behavior change
	Implementation challenges	Micro level factors
		Meso level factors
		Macro level factors
	Adoption of mHealth technologies in a specific young population	The need for training of end users
		The need for design to facilitate uptake and match social context or peer expectations
	Codesign and tailoring	Importance of codesign
		Tailoring to end user needs

^a^mHealth: mobile health.

**End user experiences of mobile health (mHealth; theme).**

Mobile health (mHealth) functionality to support self-management (subtheme)

- “I used the symptoms, triggers, and notes, cause—because with the symptoms, it can—it pretty much tells how—like what I’m feeling at that time like throughout the day and the triggers is like if I have a flare up or, uh, an attack or—then it’ll—it’ll help, it’ll show like what—what caused it in the notes because it just—I can just put down everything that happened throughout the whole day.” Carpenter 2016, *page 515, column 2* (unequivocal)
- “Like it—it really did help me out, um, and to know about the progress of my-of my asthma...it let me like know more of how my asthma was going during the weeks and—and days.” Carpenter 2016, *page 513, column 2* (unequivocal)
- “The triggers, um, I thought it was good because it would help you keep track of like what triggered it before, so you would know to stay away from it or stay indoors if it’s like a certain type of plant blooming or something. And it would help you, uh, remember that for the future years, so you could, um, remember to stay away from it.” Carpenter 2016, *Page 514, column 1* (unequivocal)
- “The chart, cause I can like sc-, I can watch it, I can scale my asthma and I can see if it’s worse or if it’s getting better, or if it’s really serious I need to do something about it, it helps me. Um-hum.” Carpenter 2016, *page 514, column 1* (unequivocal)
- “And I always remember to take my medicine easier with this app so I think that will help out. Because if I could continue to take my medication on sort of, uh, a consistent flow it makes it easier. And so overtime, I think it will help me control my asthma.” Carpenter 2016, *Page 513, column 2* (unequivocal)

mHealth functionality to support person-centered clinical encounters (subtheme)

- ‘‘They’ll [doctors and nurses] be able to know exactly what is happening.’’ Gibson 2010, *page 349, Table 3* (unequivocal)
- “I think that was good...so like if your doctor just wonders how you’re doing when he doesn’t see you, you could, you could send him the chart and he could see how you’ve been doing.” Carpenter 2016, *page 515, column 1* (unequivocal)
- “I could give it to my school if there’s a problem with my asthma, they can say, ‘Oh, well she did send us this document saying that she has asthma, so we need to let her take her medicine,’ so that’s a good thing.” Carpenter 2016, *Page 515, column 1* (unequivocal)

##### mHealth Functionality to Support Self-Management

The functionality of mHealth technologies was perceived as supporting young people’s self-management of a range of NCDs including asthma, diabetes, and cancer. Specifically, the functionality offered by mHealth technologies assisted young people in managing their conditions in a number of different ways. This included the following:

- monitoring their health status and symptom triggers via graphical charting [33] and sign or symptom awareness using self-checks [33,37,39]
- improving their comprehension and understanding of their health condition [34]
- providing reminders about medication adherence [33]
- providing ready access to automated tailoring of personal health information related to the management of their condition(s) [33]
- providing relevant information, support, and reassurance about planning for emergencies and safety issues through prompting timely communication with health professionals [35,37,39]

##### mHealth Functionality to Support Young Person-Centered Clinical Encounters

The functionality of the mHealth technologies supported a young person-centered clinical encounter by enabling accurate and immediately available clinically relevant personal data at a consultation [30], providing a record of clinical health information to treating practitioners (portability and accuracy of data over a cumulative period of time) [33], and enabling end users to direct the focus of the clinical encounter [37].

#### Theme 2. Acceptance of mHealth Technologies

End users’ acceptance of mHealth technologies was related to two subthemes: (1) technical capability (usability; how it’s working now and how they perceived optimization) and (2) acceptability and feasibility (Textbox 2).

##### Perceptions of Technical Usability

Users identified technical aspects of the mHealth technologies that affected usability and made suggestions for optimization or improvement as it related to implementation at scale.

Whereas mHealth technologies were perceived as useful to supporting their health needs [30,35], especially for tracking functions such as data logging, dose calculation (insulin), and for agenda setting (identifying and remembering what to discuss at appointment in the context of diabetes) [30], participants also identified the need for specific technical adjustments to better support management of their condition(s) [30,35]. This included bypassing the need for accessing SMS text messaging via an Internet browser on the mobile phone; however, end users preferred a capability to use direct SMS text messaging. Furthermore, end users also reported a preference for having a download availability of the software for use directly on their own mobile phones [35].

##### Perceptions and Experiences Around Acceptability and Feasibility

Users identified characteristics of mHealth technologies that aligned with their preferences for disease management support, specifically apps that were intuitive (self-explanatory and simple to understand) and provided practical self-management information that was immediately usable [30,31,35].

**Acceptance of mobile health (mHealth) technologies (theme).**

Perceptions on technical usability (subtheme)

- “The Diamob app didn’t work at the end of the project. The glucometer with Bluetooth worked, but batteries ran out of power quickly.” Froisland 2012 *ePub* (unequivocal)
- Overall, reviewers indicated that the apps were worth trialling but a few felt improvements or amendments were needed before regular use. Ashurst 2014 *ePub* (credible)
- “But what is cumbersome is that you have to access that Internet browser on the mobile. I would prefer to send normal SMS on the phone...that would make it even easier if you could access it using the usual SMS [on the phone].” Froisland 2012 *ePub* (unequivocal)
- “I think it is a lot easier to understand and to have it explained when I can see things.” Froisland 2012 (unequivocal)

Perceptions and experiences around acceptability and feasibility (subtheme)

- "I think most people just don’t want to do them [peak flows]. And you don’t want to have to—because first, you have to, you know, use it. You have to use it three times and you really start coughing, hacking after you’ve used it. Most people don’t like peak flows. And then in addition to actually having to do the peak flow, you—if you want to see how you’re doing really, you have to document it." Carpenter 2016, page 515, column 1 (unequivocal)
- Adolescents were able and willing to make adjustment to their routines to accommodate mASMAA and became accustomed to interactions with mASMAA easily (“You get used to it and it becomes routine”; “I feel like it becomes normal, just like...an instinct to do it”) Rhee 2014, *page 67, column 2* (unequivocal)
- “It is more about those messages and the information. It has been practical advice, easy to understand, simple facts that are very nice to know. It is better to have it in such small portions instead of reading a lot of information, then everything is poorly read and poorly understood. I liked the way the information was given.” Froisland 2012 *ePub* (unequivocal)
- Reviewers’ felt the easiest to use apps were self-explanatory and simple to understand. The other apps were also considered easy to use but with some suggestions to improve the user interface. Ashurst 2014 *ePub* (credible)

Whereas some features were reported as not relevant or acceptable (eg, a requirement to record peak flow for asthma management) [33], the use of mHealth technologies was still considered useful and feasible as end users were able to adapt to and accommodate mHealth technology into their routines [39].

#### Theme 3. The Importance of Codesign

End users identified the critical importance of codesign of mHealth technologies, which included subthemes based on intra and extra-personal factors considered important to end users [30] (Textbox 3).

##### Intrapersonal Factors

Competing time demands and inadequate knowledge of condition-specific triggers and value judgments (such as a perception of already adequate self-management) [33,34] were cited as factors that needed to be considered in mHealth technology codesign.

##### Extra-Personal Factors

Capacity for tailoring design and making technology more broadly acceptable for end users were important considerations. Understanding disease-specific requirements and young people’s needs around the use of technology for self-management [30] were deemed important, including design considered within the context of their specific peer or social setting [34].

#### Theme 4. Perceptions of Benefit

End users perceived benefits in the use of mHealth technology that included the subthemes of self-efficacy and empowerment (Textbox 4).

##### Self-Efficacy

End users indicated that mHealth technologies were beneficial and positively influenced their internal sense of control, consistent with improved self-efficacy [34,37,39].

##### Empowerment

mHealth technologies were perceived by end users as empowering their NCD self-management skills and knowledge. This was perceived as resulting in increased confidence and more positive perceptions about their ability to better manage their lives [33,35] through improving their knowledge and accessibility to health providers [34].

**The importance of codesign (theme).**

Intrapersonal factors (subtheme)

- “I really don’t know what my triggers are, so I really didn’t use it that much.” Carpenter 2016, *page 514, column 1* (unequivocal)
- “Because, like my asthma is well-controlled, so like a lot of the stuff here I don’t really need, but maybe like other people who have it worse will like probably need it more.” Carpenter 2016, *page 515, column 1* (unequivocal)
- “[...] one participant noted that she was too busy to use an asthma app.” Carpenter 2016, *page 515, column 1* (credible)

Extra-personal factors (subtheme)

- [...] much importance was placed on app design (not necessarily development) by diabetic peers because of a mutual understanding of the needs, condition and experiences in order for the apps to offer the most accurate features and details. Ashurst 2014 (credible)
- Most adolescents in the study felt in charge of their own life, however they talked about acceptance as an important factor. Acceptance of own disease and treatment and also acceptance from important others like friends to treatment while in different social settings. Froisland 2015, *page 545, Table 1* (credible)

**Perceptions of benefit (theme).**

Self-efficacy (subtheme)

- “[...] adolescents reported increased independence during the trial, as indicated in their improved self-management (e.g., taking medications) without parents’ prompting.” Rhee 2014, *page 68, column 2* (credible)
- ‘‘I felt in control and I liked that you could see if your temperature had improved.’’ Gibson 2010, *page 349, Table 3* (unequivocal)
- The direct contact with those they trust was reported as important. To know that they got an answer back, gave a feeling of acceptance and to be paid attention to. Froisland 2015, *page 545, Table 1* (credible)

Empowerment (subtheme)

- “It has been pretty good to know that if I have an issue, then I can just send a message...Instead of calling Mom or Dad and ask them to call [the physician], and when they have the answer it might be an answer to something I was not wondering about.” Froisland 2012, *page 513, column 2* (unequivocal)
- “It kind of keeps me to where I can see what I’ve done, instead of it just being in my mom or my doctor knowing how far I’ve come, where—if I’m getting better or worse, if I’m normal for myself or anything, I can kind of keep myself in check.” Carpenter 2016, *page 516, column 1* (unequivocal)
- Positive response from people who know the disease is important to feel empowered. The SMS application increased the possibility for response directly from their health care professional. Froisland 2015, *page 545, Table 1* (credible)

### Implementers’ Experiences and Perspectives

#### Theme 1. mHealth Characteristics That Support Young People’s Management of NCDs

Implementers identified multiple components of young people’s NCD management that can be supported by mHealth technologies (Textbox 5). Three subthemes emerged: functional aspects of design that support end users’ management, technical characteristics that support clinicians’ delivery of clinical care for young people, and how mHealth can support positive health behavior change.

##### Functional Aspects of Design That Support End Users’ Management

Implementers identified a range of design features that were perceived to support end users’ management of their conditions. These included the following:

- tracking side effects and symptoms for clinical management [36,40,41]
- focusing the agenda for clinical appointments [36,40,41]
- reminders for medication adherence and to overcome supply problems [36,41]
- enabling bilateral communication between end users and clinicians [32,36,40]
- overcoming communication deficiencies [32]
- habituation of components of self-management (medication management and adherence [40])
- providing alerts for end users and their clinicians about deteriorating health conditions [40]
- remote technology enabling social connectedness and access to health support (motivation, coaching, and providing information to their treating physician) [32,41].

##### Technical Characteristics Can Help Their Delivery of Clinical Care

Implementers identified several technical features that they believed would assist their delivery of clinical care and optimize their engagement with end users, such as communication reminders (use of medicines and low peak flows) and focusing clinical encounters through more efficient preparation [36,40].

##### mHealth Can Support Positive Health Behavior Change

Implementers perceived mHealth technologies to positively influence end users to independently manage their condition and to facilitate positive health behavior change [32,36,40] through independent communication [32], age-related appeal [36], and providing positive feedback to end users (eg, improved asthma tracking, reminders for medication use and refills, peak flow assessment, and communication to health professionals) [40].

**Mobile health (mHealth) technology characteristics that support young people’s management of noncommunicable diseases (theme).**

Functional aspects of design that support end users’ management (subtheme)

- “This way you can look back over the previous 4 weeks or 3 months and focus on questions such as—“you scored sleep a 2 here, what was happening at the time that made it so unsettled?” It should help parents to be more productive in giving the information we need.” Simons 2016, *page 9, column 1* (unequivocal)
- “Participants saw the potential for RMT to provide the ability to easily monitor symptoms, chart them over time, and identify any patterns or unusual behaviors. This would increase people’s knowledge, self-awareness, and understanding of and confidence in dealing with their condition.” Simons 2016, *page 9, column 1* (credible)
- "The difficulties I come across, [are that] young people are on medication and they tend to run out at the end of the month and their behavior will go sky high, and it will take them a week to get all the medication back into their system. I think it would be really useful if somewhere in the app, say when they’re...near the end [they receive a message saying] 'You need to put in a request for repeat prescription.'" [HCP, Site 3] Simons 2016, *page 10, column 1* (unequivocal)
- ‘‘ … teenagers are busy and communication is limited and I think using technology will improve communication. They’ll listen more. I mean, I think they read their texts, you know, and I think reading a short text is much more beneficial and reminder systems on an everyday, I mean, doing something the same way for 2 weeks makes it a habit.’’ Schneider 2016, *page 156* (unequivocal)
- “He has great help from the synthetic speech and he is markedly disturbed when it doesn’t really sound like he wants it to.” Buchholz 2013, *page 92, column 1* (unequivocal)

How technical characteristics of mobile health (mHealth) can help their delivery of clinical care (subtheme)

- Clinicians felt that use of the app could lead to a better medical appointment both in terms of efficiency, patient-centered care, and decision making. Multiple clinicians expressed data security concerns (eg, insecure email) or differed in their preference for information delivery method [...] Geryk 2016 *ePub* (credible)
- Multiple clinicians mentioned that appointment noncompliance is a problem, one stating that “[a]ny extra reminder that families have that they have an appointment I think is helpful.” Geryk 2016 *ePub* (unequivocal)

How mHealth technology can support positive behavioral change (subtheme)

- ‘‘I mean if everything is going well, you could give them sort of positive feedback just like: ‘‘Hey, keep up the great work.’’ If not, you could be like: ‘‘Are you taking your controller?’’ Schneider 2016, *page 158* (unequivocal)
- Clinicians generally had positive things to say about the apps as a self-management tool to help parents and adolescents including the following: “hands-on” and provides a “more interactive or fun way to check on their asthma.” Geryk 2016 *ePub (credible)*

#### Theme 2. Implementation Challenges

Important challenges to implementation of mHealth technologies were experienced or perceived by implementers as extending across multiple levels of the health care system. This aligned with three subthemes: challenges at the clinical level (micro), challenges at the service delivery level (meso), and challenges at a systems level (macro; Textbox 6).

##### Micro Level Factors

Factors identified as barriers to implementation at the clinical level included accuracy of health indicator monitoring [36] and a limitation of task-specific capability for specific health conditions [32].

##### Meso Level Factors

At the organizational level, key factors identified as barriers included the internal regulatory environment of organizations [38], resource allocation (remuneration and funding) [40], issues with integration into the current work flow [38,41], organizational climate and readiness for change [38], and interoperability with existing information and technology infrastructures [41].

##### Macro Level Factors

At the systems level, health information security and national or jurisdictional electronic health (eHealth) regulatory frameworks were highlighted as key challenges to implementation of mHealth technologies [36,40].

#### Theme 3. Adoption of mHealth Technologies in a Specific Young Population

Implementers perceived the need for mHealth to be adaptable or tailored for vulnerable populations, referring specifically to young people with cognitive and communicative disability. Two subthemes emerged: (1) the need for training of end users and (2) the need for design to facilitate uptake and match social context or peer expectations (Textbox 7).

##### The Need for Training of End Users

In a single study, Bucholtz et al [32] identified that specific training of end users is required to facilitate better uptake or adoption of mHealth technologies in this specific population.

##### The Need for Design to Facilitate Uptake and Match Social Context or Peer Expectations

Design to facilitate adoption included a focus on mHealth technology supporting end users “blending in” and a capacity to streamline function with their existing technology (eg, software installed on end users’ own mobile phones). Additional considerations were devices that were physically easy to handle, hardware designed to meet specific end user needs (eg, texting with symbols and speech synthesis), and devices that fit well into end users’ daily routines.

#### Theme 4. Codesign and Tailoring

Implementers perceived specific characteristics of mHealth technologies that they considered important to support end users’ management of NCDs. Two subthemes emerged: (1) the importance of codesign and (2) tailoring to end user needs (Textbox 8).

**Implementation challenges (theme).**

Technical features as barriers to implementation at the clinical (micro) level (subtheme)

- This [technical asthma trigger] feature was more often criticized by parents and clinicians because of its lack of long-term monitoring and feedback capabilities. One clinician expressed the opinions of other participants when stating, “I don’t know what you’d [do] with it. Other than just be aware of it.” Geryk 2016 *ePub* (unequivocal)
- “Basically he seems to think it’s good but he’s frustrated because he thinks...he has very high expectations and to this point he doesn’t feel they have been met” Buchholz 2013, *page 91, column 1* (unequivocal)

Organizational level (meso) barriers to implementation (subtheme)

- ‘‘The biggest thing is...a time issue, lack of reimbursement...for adding additional duties.’’ Schneider 2016, *page 157* (unequivocal)
- “We see young people with severe mental health problems, including suicidal ideation, and I’m not sure it’s ideal for this group...Most self-harm is dealt with by family support workers and schools, and they are always looking for additional resources and tools to help with it.” Owens 2016, *page 7, column 1* (unequivocal)
- “The general perception within the team is that using TeenTEXT is too much of an extra burden on top of our existing workload.” Owens 2016, *page 6, column 2* (unequivocal)
- “The organisation doesn’t give clinicians any leeway. We need permission to try anything new and there are so many hoops to jump through before that happens.” Owens 2016 (unequivocal)

System level (macro) barriers to implementation (subtheme)

- Clinicians felt that use of the app could lead to a better medical appointment both in terms of efficiency, patient-centered care, and decision making. Multiple clinicians expressed data security concerns (eg, insecure email) or differed in their preference for information delivery method [...] Geryk 2016 *ePub* (credible)
- “Oh, I would love to do it by electronic means. The problem is that then you run into all the HIPAA problems.’’ Schneider 2016, *page 157* (unequivocal)

**Adoption of mobile health (mHealth) technologies in a specific young population (theme).**

The need for training for end users for some conditions and settings to facilitate adoption (subtheme)

- “It has been easy to handle for him...it has been easy also in terms of making adaptations (for the helper).” Buchholz 2013, *page 91, column 2* (unequivocal)
- “This is an aid that would be of help for a lot of people. I have many colleagues with clients who would need something similar maybe particularly adolescents that are becoming adults.” Buchholz 2013, *page 92, column 2* (unequivocal)
- “Exciting a little more up to date...modern...or she would never have accepted it.” Buchholz 2013, *page 91, column 1* (unequivocal)
- “Yes because if this software was installed in the regular phone I think she would use it more.” Buchholz 2013, *page 91, column 1* (unequivocal)

Need for codesign to facilitate uptake and social currency (subtheme)

- “It’s important to find a situation where you really see the need of being able to text or a person you need to contact where a regular phone call won’t work.” Buchholz 2013, *page 92, column 1* (unequivocal)
- “Yes, it was very abstract I think so when we could show him something more concrete he grasped it better.” Buchholz 2013, *page 91, column 2* (credible)

**Codesign and tailoring (theme).**

Importance of codesign: implementers identified the importance of working collaboratively with end users to optimize functionality (subtheme)

- “She has great use of them and we have built upon her interests so she can easily reply to a text and she can also send a pre-designed text.” Buchholz 2013, *page 93, column 2* (unequivocal)
- One clinician brought up the benefits of using the feature for “engaging with them [patients]” including jointly inputting information into the plan and/or discussing what patients have previously input to ensure they are getting the correct guidance, especially regarding emergency situations. Geryk 2016 *ePub* (credible)
- “I like the fact that the messages are written by them, so they’re supporting themselves... This fits with what we currently do, which is try and give them a sense of control.” Owens 2016, *page 5, column 1* (unequivocal)

Need for technologies to be tailored to end user’s needs and contexts (subtheme)

- “Yeah it’s like that. He has started to use it more for face to face communication...not just the text-messaging function but more as a communication device.” Buchholz 2013, *page 94, column 1* (unequivocal)
- “I think most of them engage in devices like this for entertainment, right? And so you want to have something that provides them an educational opportunity, um, but also something that they – they won’t get bored with.” Geryk 2016 *ePub* (unequivocal)
- ‘‘Don’t forget to pretreat before you go out for soccer practice, or football practice,’’ specific for that patient’s sport I think would be even more, you know, something that’s specific for that patient.’’ Schneider 2016, *page 158* (unequivocal)

##### Importance of Codesign

Implementers identified the importance of working collaboratively with end users to optimize functionality requirements as part of the early phase of development of mHealth technologies [32,36,38].

##### Tailoring to End User Needs

Implementers identified the need for the design of mHealth technologies to be adaptable to end users, providing for tailored age-relevant design, content, and functionality [36,40], as well as meeting condition-specific requirements [32].

### Policy and Practice Recommendations and Implications

On the basis of our evidence meta-synthesis, we derived five key recommendations and described the associated policy and practice implications (Textboxes 9-13). The use of mHealth in management of young people with chronic NCDs can support self-management and drive meaningful change in contemporary health ecosystems. However, identifying and resolving implementation challenges is critical to enabling sustainable scaling-up of mHealth solutions. These recommendations should help to inform appropriate resource design, evaluation, and implementation in a way which all users will find acceptable and which health systems will find sustainable.

**Recommendation 1 and implications.**

Recommendation: Mobile health (mHealth) technologies should be considered as a potential strategy or solution to enable self-management, to improve clinical encounters, and to encourage positive health behaviors in young people with chronic noncommunicable diseases (NCDs).

Implications:

- mHealth should be considered by consumers and stakeholders involved in the delivery of care as a complement to existing health care options, as a means to enhance care delivery and efficiency and to integrate into care pathways 
- To achieve this outcome, it is important to clearly identify end users’ needs and also to identify where and when in a young person’s care pathway mHealth technologies could meaningfully affect capacity for self-management, improve clinical encounters, and influence positive health behavior
- Policy makers need to respond to the momentum around mHealth by considering current care pathways and support systems and identifying opportunities for integration of mHealth technologies to optimize cocare; to facilitate location-based care; and drive quality, safety, and efficiency in care delivery

**Recommendation 2 and implications.**

Recommendation: Design of mHealth technologies for young people with chronic NCDs should be a collaborative process involving partnerships with multi-stakeholders (eg, young people, health professionals, digital technology designers, service delivery, and policy makers) to achieve meaningful codesign and to inform appropriate implementation approaches. 

Implications:

- A collaboration of relevant stakeholders needs to be engaged from inception and at all stages through planning, developing, testing, implementing and through continuous cycles of improvement (formative evaluation) for mHealth technologies 
- Importantly, different stakeholders may be needed at different stages and these stakeholders should be explicitly identified to align with requirements at each stage
- From inception, processes should be informed by contemporary evidence and an appropriate implementation science framework
- The outcome of this collaborative and evidence-informed approach should ensure that mHealth technologies have social currency and are contemporary, relevant and useful to young people

**Recommendation 3 and implications.**

Recommendation: mHealth technologies for chronic NCD management in young people need to have functional capabilities that allow for tailoring to end users’ preferences and person-centered needs.

Implications:

- Implementers need to undertake formative evaluations of mHealth technologies across the development and implementation stages in partnership with young people to ensure that functionality is responsive to their end user needs, including changing developmental and NCD needs
- These formative evaluation outcomes need to direct iterations of mHealth technologies

**Recommendation 4 and implications.**

Recommendation: Implementation initiatives must consider whole-of-system readiness to adopt mHealth technologies. The use of contemporary mHealth toolkits for planning and scale is advisable [67].

Implications:

At a health systems (*macro*) level, it is necessary to consider system readiness to support implementation and adherence. This requires identifying gaps and opportunities across the system to support implementation, including

- current policy or strategy platforms
- workforce capacity building initiatives and priorities
- infrastructure and human resourcing
- strategic cross-sector partnerships
- alignment with existing policy, technological, legal, and regulatory frameworks. Compliance with information and communication technology regulatory frameworks is imperative

At the service delivery (*meso*) level it is necessary to consider

- organizational readiness for change (eg, culture, change management leadership, executive support, and technophobia)
- seamless integration of mHealth into existing and planned workflow
- business modeling to capture value, cost effectiveness, and sustainability
- interoperability with existing information and technology systems

At the clinical (*micro*) level, implementers need to jointly assess, in partnership with health providers and end users, the desired functionality, required accuracy of data capture, and security associated with the use of proposed mHealth technologies

**Recommendation 5 and implications**

Recommendation: Implementers of mHealth technologies must undertake continuous cycles of improvement to maintain technical and functional optimization. The use of contemporary digital health monitoring and evaluation guidance is advisable [68].

Implications:

- Given the rapidly changing landscape of mHealth technologies, continuous technical updates are needed to address changes (to maintain platform compliance and security)
- Planned review cycles are necessary to allow for iteration and optimization of content and functionality based on analytics data
- A governance framework needs to be developed in advance of implementation, with the aim of addressing project management and guiding these review cycles
- Dedicated resourcing is required to implement such a framework

## Discussion

### Principal Findings

This systematic review extends our understanding of users’ experiences and perspectives of mHealth for chronic NCDs management in young people and highlights the specific enablers and barriers to implementation. The clear evidence of benefit for the use of mHealth technologies by young people for education, monitoring, and the self-management of their chronic NCDs often fails to sustainably translate into real-world settings, consistent with reports that “... *benefits can only spring from effective implementation that credits interaction with human and organizational factors* ” [69]. Our evidence synthesis provides novel insights to inform and guide actionable policy and practice recommendations on “how” we can implement mHealth technologies to better support young people’s management of their chronic NCDs. The key findings from this evidence synthesis also show both complementary and unique perspectives on the use of mHealth for chronic NCD management in young people. Collectively, mHealth technologies were perceived by users as supporting young people’s self-management across a range of chronic NCDs including diabetes [30,34,35], cancer (chemotherapy symptom management) [31,37], asthma [33,39,40], cognitive and communicative disabilities [32], chronic self-harm [38], and ADHD [41]. No studies were identified that specifically examined persistent musculoskeletal pain.

Complementary perspectives on the use of mHealth technologies to enable young people’s management of NCDs were evident for a number of themes and subthemes. These included codesign of mHealth technologies; functional and technical aspects of mHealth technologies that were person-centered and which aligned with young people’s current technology use (habits, routines, and preferences); and which supported the delivery of clinical care and positive behavior change. The benefits of mHealth use were uniquely perceived by end users (young people) as empowering them to more independently manage their chronic health conditions.

**Figure 2:**
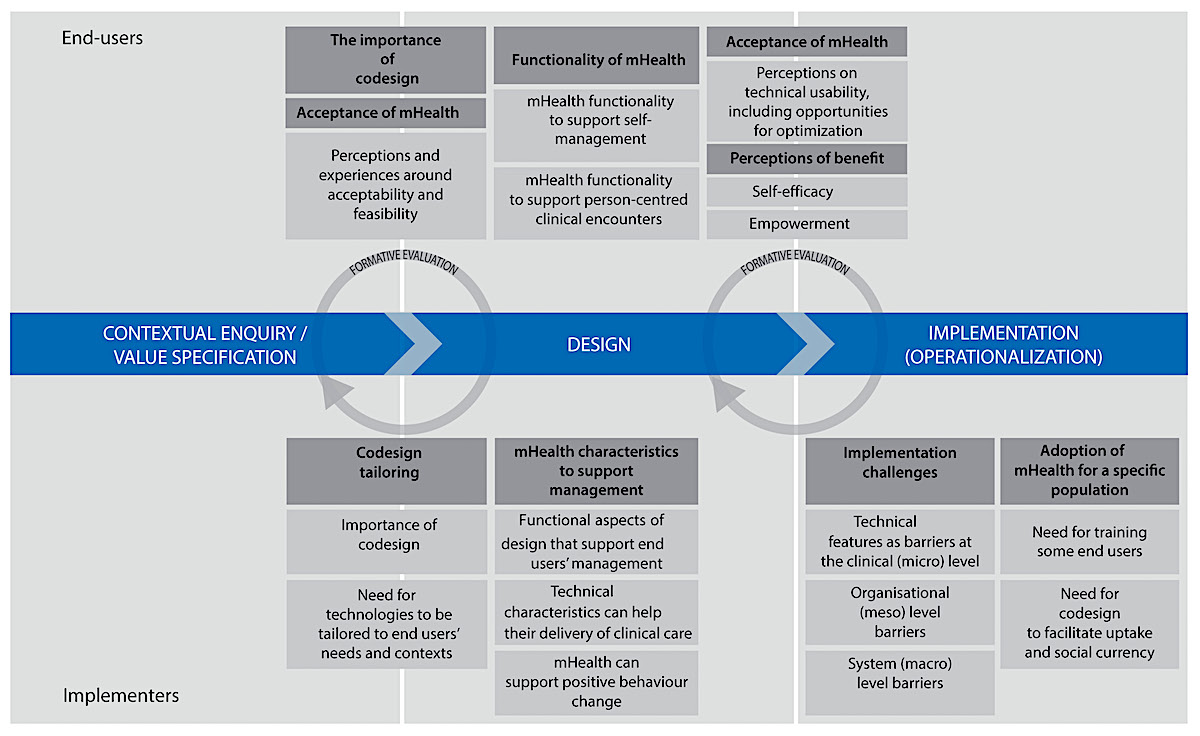
A representation of the review findings is mapped against relevant elements of the Holistic Framework and applied here as a theoretical underpinning to guide our discussion. Themed categories for end users are represented above the blue line and implementers below. Implementation phases are represented by the central blue line, which indicates a left to right movement showing the continuous and iterative cycles of mobile health (mHealth) development. This includes phases from predevelopment (enquiry or value specification), to design and implementation (operationalization), with formative feedback guiding iterations of mHealth technologies. Note, both complementary and unique user perspectives are evident.

Implementers (specifically clinicians) perceived a great benefit in mHealth affording access to clinical data during consultations and as an enabler to support person-centered clinical encounters. Barriers to the uptake or adoption of mHealth technologies were uniquely identified by implementers as representing “whole of system” (multi-level) factors, including at the clinical level (micro factors), at the organizational level (meso factors), and at the systems level (macro factors). Implementers also identified the need for specific design considerations for mHealth apps for a vulnerable population.

These complementary and unique perspectives highlight both the interdependencies and complexities encountered by different users interacting with a rapidly evolving digital health ecosystem. To interpret our findings and make meaningful recommendations for policy and practice, the use of a design and implementation framework that is plural and pragmatic helps to address such complex interdependencies between human characteristics (users), digital technologies, and health systems. Figure 2 shows the application of such a framework to our synthesized findings (darker shading indicates themes and lighter shading, subthemes) [69]. This Holistic Framework developed by van Gemert-Pijnen and colleagues [69] has been widely used to guide the design and implementation of eHealth technologies in chronic care management [16]. The framework allows for an inherently fluid, iterative, and cyclical nature of design, implementation, and evaluation of digital technologies. We focused on key domains relevant to our findings (contextual enquiry and value specification, design and implementation [operationalization]) [69]. Given the significant overlay between contextual enquiry and value specification in our data, these were collapsed into a single domain.

### Complementary Users’ Perspectives on the Importance of Codesign

Codesign emerged for all users as a fundamental design principle and enabler to the uptake of mHealth technologies. The triangulation between user group perspectives is reflected in the mirroring of themes on codesign, as shown in Figure 2. These complementary perspectives related to (1) the “contextual enquiry and value specification” domain and (2) the “design” domain. For this reason, codesign is shown in Figure 2 as overlapping both these domains. A formative evaluation loop guides iterations to mHealth technologies during this developmental phase; a step also identified in the primary studies as an important component of mHealth development. Involving end users and other stakeholder user-groups was perceived as critical to ensuring a clear understanding of (1) what the end user wants and needs to best support their self-management (user-friendly, acceptable, meaningful, and safe) and (2) how mHealth technologies could be optimized to meet person-centered needs and support behavior change. Using participatory models of codesign to jointly develop digital technologies that is meaningful to end users, aligns with current recommendations for development and implementation of digital technologies [16,18,69]. In a recent study published outside of our search dates, user-centered codesign principles were effectively applied to improve usability (easy to use, easy to understand, efficient to complete, and acceptable) of a real-time mHealth app for adolescents self-managing cancer pain [14].

Clarity was also deemed important by users around identifying who the required stakeholders would be, what specific roles they would undertake, and at what stages they would be needed. These findings are consistent with recommendations from a recent systematic review of mHealth for NCD management indicating a need for explicit identification of relevant stakeholders as a mechanism to help make sense of eHealth systems for users, to specify mHealth purposes and benefits, and to establish their value, including identifying factors promoting or inhibiting engagement and participation [70].

Contextual enquiry allows for identification of factors relevant to guiding mHealth design that is acceptable and feasible for end users; a theme that emerged from users reported in the primary studies in our review and more widely reported by others as a critical design factor [1,70,71]. Contextual factors from our review included value specifications such as the intended use of technology (self-management), the nature of the condition (eg, NCDs, disease status, and level of impairment), the target population (young people), functional requirements (eg, monitoring, medication titration, tracking, decision-support, goal-setting, and cocare), and the care setting (eg, home, school, work, and hospital). Similar factors have been identified in recent systematic reviews of mHealth technology use in NCDs [1,18,27]. Implementers’ values were further reflected in their perspectives on the importance of the tailoring capabilities of mHealth to meet end users’ specific condition needs. Organizational needs did not emerge in this review as a key codesign value specification, although contemporary guidance on mHealth technology would suggest this is a critical preimplementation factor [67,69].

### Users’ Perspectives on the Importance of mHealth Design Characteristics

Emerging evidence supports use of mHealth for self-management to facilitate clinical interactions and to encourage positive health behaviors [16]. To promote use and adherence, mHealth design needs to reflect meaningful functionality for end users [1,4,15,27] and to make sense within the context of their daily lives [16]. Our findings support these recommendations with mHealth functionality identified as a critical design factor by both user groups (Figure 2).

End users’ perceived functional characteristics of mHealth technologies as helping their self-management adherence, including self-tracking, condition self-monitoring (condition status and medication), self-observations providing for early warning of condition flare-ups, self-reflection, improving their understanding of their condition, and providing reassurance by facilitating contact with their health professionals. Implementers’ perspectives similarly recognized meaningful functionalities could assist adherence by leveraging off young people’s habitual use of mHealth technologies. Functionality that extended reach to young people in remote settings, or to those with low accessibility was also perceived by implementers as important; an issue highlighted by us in a study of the gaps and needs of young people with persistent musculoskeletal pain [2] and consistent with health policy in nations with large care disparity gaps created by geography, such as Australia and Canada.

Functionality characteristics that enabled person-centered care was identified by both user groups as important, including features that focused end users on their condition status and helped them prepare for clinical encounters. From the implementer perspective, technical capabilities were perceived as enablers to supporting their delivery of clinical care. While protecting patient privacy, similar technical capabilities that supported person-centered care by facilitating bilateral communication and which helped the end user focus on the purpose of the clinical encounter were perceived as important. Consistent with these findings, systematic review-level evidence indicates that person-centered care is a key enabler to adoption and adherence of mHealth technologies for self-management [16,18]. This person-centered focus is also central to recommendations from contemporary health policy across all settings and economies [67,68].

Implementers described mHealth technologies as helpful in supporting behavior change for young people with NCDs. For example, through sustained engagement of young people by monitoring of their health condition and by providing positive feedback as reinforcement for behavior change. Here, mHealth technologies may be utilized as a catalytic tool for driving sustainable management of NCDs [67,68]. However, perceptions and actual outcomes around behavioral change do not necessarily align. More effort and focus is required to understand how mHealth technologies can be used to effect meaningful, sustained behavior change [27,72]. This emerging area requires more than pilot or feasibility studies, arguing for more appropriately designed trials, longer term evaluation, and real-world, population-based health monitoring [68,69,73].

### Users’ Perspectives on mHealth Technology Implementation Challenges and Solutions

Technical issues associated with real-world use of mHealth technologies impact usability and wider acceptance (end users), scaling-up, and sustainable implementation (implementers; Figure 2). The need to address recognized technical issues and to optimize mHealth technologies in the “readiness” phase of implementation highlights the critical role for rapid, continuous cycles of evaluation (formative and summative evaluation). Linking design refinements to improve end user experience and to help drive adoption and uptake (ie, implementation “success”) emerged as important for both user groups in our review. Judging “readiness“ and “success” can help mitigate against implementation challenges, and we have derived such a system-level framework that is described comprehensively elsewhere [23].

From the end user perspective, mHealth apps that are readily accessible and downloadable onto young people’s current mobile devices is an example of one such “readiness” lever [2,8], especially if apps align with end users’ habitual routines [16]. Implementers also highlighted the need for accurate disease monitoring and task-specific capabilities to support young people with unique NCD requirements. These perspectives again emphasize the importance of upstream “readiness” contextual enquiry and value specification as integral to effective codesign and to supporting successful downstream implementation efforts [23].

Although contemporary health policy reform agendas articulate the need for innovative use of mHealth for NCD management [7,26,74], currently, very limited processes and frameworks exist to guide the development and implementation [17,18,75,76]. This challenge resonates with the findings of our review. Many studies consisted of pilot projects or small-scale implementations with evidence of feasibility and acceptability (as per their study aims), however, without extensive consideration of the implementation frameworks needed for building scale. Even with the application of theoretical frameworks to mHealth technologies to gauge scalability (eg, the use of normalization process theory; person-centered design and participatory methods of intervention development), significant barriers to implementation can still stymie uptake [38]. These same mHealth technology implementation challenges are articulated in reviews of older populations with NCDs [18,72]. In the latter review by Matthew-Maich and colleagues [18], successful implementation of mHealth required addressing factors across the whole of health systems. Our review found similar “whole of system” factors, including at the *micro* level (technical factors); at the *meso* level (organizational, culture, climate, environment, health workforce needs, work flow disruption, technophobia, natural fit for population and health condition, and funding models); and at the *macro* level (regulatory frameworks, governance, and flexibility; Figure 2). These multilevel barriers emphasize the critical importance of taking a system-wide approach to supporting implementation (for comprehensive reviews on implementation, see Briggs et al) [77,78]. Such an approach involves the systematic identification of “readiness” for implementation, as well as postimplementation evaluation of “success” [23]. This approach aligns well with the Holistic Framework we have adopted here for the specific embedding of mHealth technologies within complex health ecosystems [16].

### Moving mHealth From Promise Into Policy and Practice

It is hard to see a future without mHealth technologies as a complement to a rapidly evolving health care ecosystem. Digital disruption is here. Rather than focusing on barriers and challenges, perhaps we need to seek opportunities for embedding of mHealth within existing health systems where evidence for effectiveness is already well established (eg, self-management) [16]. Further value may be derived from identifying where in health systems, health services, and clinical populations or interfaces potential synergies can be identified that provide a natural “fit” for implementing and building scale in mHealth use [72]. Here, mHealth can be viewed as a catalytic tool implemented to strengthen health systems [67,79]. In lower and middle-income countries, factors such as a lack of infrastructure, health workforces, resources, and regulatory frameworks have already driven innovative mHealth solutions; for example, using partnerships arrangements and modifications of existing mHealth technologies that can be readily and sustainably implemented [8]. Implementation guidance and enabling strategies to support mHealth initiatives more broadly is available, for example, in the mHealth assessment and planning for scale toolkit [67].

Beyond implementation, ongoing evaluation and monitoring of mobile and other digital health interventions is deemed critical to inform health policy and practice [80,81]. The World Health Organization provides guidance in this regard from the collective learning of 5 years of engagement with various international lead agencies working to strengthen their digital health deployments, develop robust evaluations, and scale up their activities nationally and regionally [68].

### Strengths and Limitations

The Holistic Framework adopted to underpin the interpretation of our review findings is based on extensive research on the uptake and impact of eHealth technologies and on models for development, implementation, and evaluation [69]. The Framework also provides a level of construct validity to our findings. Whereas consideration was given to alternate implementation frameworks [13] such as the Consolidated Framework for Implementation Research [82], technology acceptance model [83], and normalization process theory [70], none of these frameworks better satisfied the need for both an integrated whole of system approach and one specifically validated for eHealth applications.

The number of studies in this review provided sufficient data to interrogate our review questions and represented both end users and implementers. The yield was not sufficient, however, to enable meaningful sensitivity analyses to be undertaken based on criteria such as study quality, diseases, settings, or credibility of findings. Most studies used mHealth apps to support self-management and comanagement of young people with NCDs. End users included young people in our age range of interest; however, most were focused at the younger end of this range. Generalizability to other cultures and contexts was limited by the small samples and by cultural and socioeconomic specificity. Our results may not be transferable to low and middle-income economies despite almost ubiquitous use of mobile phones. This represents a critical area of research need given the widespread use of mobile technologies in such global settings and the urgent need to address NCDs through health information and health connectivity at scale [84,85]. Implementers were broadly representative of the whole of system; however, health policy makers were not explicitly identified. Although we did not include parents as implementers specifically in our search, for two [36,41] of three possible studies that included parents, their perspectives were captured within pooled implementer data. Explicit parent perspectives may provide important additional insights especially for the younger end of our age range of interest. Data on experiences and perspectives about actual or potential risk and harm associated with use of mHealth technologies were limited, although these are very important factors to consider [86].

Most studies were of short duration, posing challenges for exploring implementation effectiveness and limiting long-term evaluation of outcomes. The quality of studies was variable, and the use of reporting standards for qualitative research (such as the Consolidated Criteria for Reporting Qualitative Research) [87] was inconsistent, possibly suggesting a high risk of bias. This raises issues of confidence about internal validity and trustworthiness, making the data extraction, interpretation, and the confidence in evidence more complex. The confidence of reported findings could be readily addressed with the use of a reporting system such as Confidence in the Evidence from Reviews of Qualitative Research [88]. Another quality indicator that was insufficiently met for most studies was the positioning of the researcher within the research, arguing again for improved reporting against standards. Some studies also provided secondary data interpretation without explicit quotations to support their interpretation, suggesting potential researcher bias. Study designs that better align with the rapid evolution of mHealth technologies are required as randomized trials are expensive, slow, and do not accommodate the dynamic nature of digital technologies, issues also highlighted by others [15,73].

### Conclusions

Our evidence meta-synthesis revealed both complementary and unique user perspectives on enablers and barriers to designing, developing, and implementing mHealth technologies to support young people’s management of chronic NCDs. mHealth technologies should be considered as a tool to enable self-management, to improve clinical encounters, and to encourage positive health behaviors. Developing mHealth technologies should involve a genuinely collaborative codesign process between end users and implementers, with the capacity to tailor and adapt technologies to meet person-centered needs. This approach will help to ensure meaningful mHealth solutions for young people, while also supporting implementation efforts. Whole-of-system readiness to adopt mHealth technologies must be considered if implementation initiatives are to be successful and sustainable. Continuous cycles of improvement are needed to maintain technical and functional optimization, ensuring that mHealth solutions remain relevant to young people. The use of contemporary frameworks that support digital health monitoring and provide evaluation guidance is advisable.

## Appendix

Multimedia Appendix 1 Preferred reporting items for systematic reviews and meta-analyses (PRISMA) checklist.

Multimedia Appendix 2 Enhancing transparency in reporting the synthesis of qualitative research (ENTREQ) checklist.

Multimedia Appendix 3 Search strategy.

Multimedia Appendix 4 Themed categories for end users’ experiences of mHealth technologies.

Multimedia Appendix 5 Themed categories for implementers’ experiences of mHealth technologies.

## Abbreviations

ADHD: attention deficit hyperactivity disorder
eHealth: electronic health
JBI-QARI: Joanna Briggs Institute, Meta-Analysis of Statistics Assessment and Review Instrument
mHealth: mobile health
NCD: noncommunicable disease
SMS: short message service
