# Supplementary material for: End User and Implementer Experiences of mHealth Technologies for Noncommunicable Chronic Disease Management in Young Adults: Systematic Review
Source: J Med Internet Res. 2017 Dec 12;19(12):e406. doi: 10.2196/jmir.8888 (PMC5743925; doi:10.2196/jmir.8888)
Supplement: Multimedia Appendix 3 [file jmir_v19i12e406_app3.pdf]

Search strategy

Total: 4046
Duplicates removed: 1193
Final total: 2853

Pubmed

Filters: Year 2007, English
Hits: 891
21/12/16

Qualitative

Qualitative research[Text Word] OR ethnology[Text Word] OR ethnological[Text Word] OR stories[Text Word] OR story[Text Word] OR content analysis[Text Word] OR content analyses[Text Word] OR ethnographic[Text Word] OR observational methods[Text Word] OR participant observation[Text Word] OR field notes[Text Word] OR experiences[Text Word] OR narrative[Text Word] OR narratives[Text Word] OR discourse[Text Word] OR process evaluation[Text Word] OR service need[Text Word] OR service needs[Text Word]OR feelings[Text Word] OR ethnopsychology[Text Word] OR focus groups[Text Word] OR focus group[Text Word] OR behavioral research[Text Word] OR behavioural research[Text Word] OR narration[Text Word] OR satisfaction[Text Word] OR dissatisfaction[Text Word] OR meanings[Text Word] OR meaning[Text Word] OR perspectives[Text Word] OR perspective[Text Word] OR perceived[Text Word] OR perceives[Text Word] OR perceive[Text Word] OR perceptions[Text Word] OR perception[Text Word] OR views[Text Word] OR view[Text Word] OR qualitative[Text Word] OR interviewed[Text Word] OR interviewing[Text Word] OR interviewer[Text Word] OR interviews[Text Word] OR interview[Text Word] OR comprehension[Text Word] OR opinions[Text Word] OR opinion[Text Word] OR expectations[Text Word] OR expectation[Text Word] OR thoughts[Text Word] OR narratives[Text Word] OR standpoint[Text Word] OR standpoints[Text Word] OR viewpoints[Text Word] OR viewpoint[Text Word] OR thematic analysis[Text Word] OR phenomenology[Text Word] OR phenomenological[Text Word] OR grounded theory[Text Word] OR grounded studies[Text Word] OR grounded research[Text Word] OR constant comparative[Text Word] OR constant comparison[Text Word] OR field study[Text Word] OR field studies[Text Word] OR field research[Text Word] OR field work[Text Word]OR biographical method[Text Word] OR theoretical sample[Text Word] OR theoretical sampling[Text Word] OR open-ended[Text Word] OR open ended[Text Word] OR life world[Text Word] OR life-world[Text Word] OR conversation analysis[Text Word] OR conversational analyses[Text Word] OR theoretical saturation[Text Word] OR thematic analysis[Text Word] OR thematic analyses[Text Word] OR action research[Text Word] OR questionnaire[Text Word] OR mixed method[Text Word] OR mixed methodology[Text Word]

Chronic non-communicable disease

Chronic[Text Word] OR long term[Text Word] OR persistent[Text Word] OR non-communicable[Text Word] OR respiratory[Text Word] OR asthma[Text Word] OR cystic fibrosis[Text Word] OR lung disease[Text Word] OR diabetes[Text Word] OR cancer[Text Word] OR heart disease[Text Word] OR cardiovascular disease[Text Word] OR pain[Text Word] OR muscular[Text Word] OR joint[Text Word] OR musculoskeletal[Text Word] OR kidney disease[Text Word] OR arthritis[Text Word] OR depression[Text Word] OR depressed[Text Word] OR anxiety[Text Word] OR anxious[Text Word] OR mental health[Text Word] OR schizophrenia[Text Word] OR fibromyalgia[Text Word] OR sickle cell[Text Word] OR disability[Text Word] OR disabled[Text Word] OR autism[Text Word] OR Asperger’s[Text Word] OR adhd[Text Word] OR attention deficit hyperactivity disorder[Text Word] OR pulmonary[Text Word] OR cardiac[Text Word] OR ischemic [Text Word] OR renal[Text Word] OR neurological[Text Word] OR osteoarthritis[Text Word] OR osteoporosis[Text Word] OR ankylosing spondylitis[Text Word] OR psoriatic arthritis[Text Word] OR rheumatoid arthritis[Text Word] OR inflammatory arthritis[Text Word] OR lupus[Text Word] OR systemic lupus erythematosus[Text Word] OR juvenile idiopathic arthritis[Text Word] OR juvenile chronic arthritis[Text Word] OR back pain[Text Word] OR neck pain[Text Word] OR stress[Text Word] OR psychological[Text Word]

Young people

young[Text Word] OR adolescent[Text Word] OR adolescence[Text Word] OR student[Text Word] OR students[Text Word] OR teenager[Text Word] OR teenage[Text Word] OR teenagers[Text Word] OR teen[Text Word] OR teens[Text Word] OR young adult[Text Word] OR young adults[Text Word] OR child[Text Word] OR children[Text Word] OR pediatric[Text Word] OR paediatric[Text Word]

mHealth

eHealth[Text Word] OR mHealth[Text Word] OR mobile application[Text Word] OR mobile applications[Text Word] OR smartphone[Text Word] OR cell phones[Text Word] OR mobile game[Text Word] OR text messaging[Text Word] OR mobile phone[Text Word] OR mobile health app[Text Word] OR mobile health application[Text Word] OR digital technologies[Text Word] OR digital technology[Text Word] OR mobile[Text Word] OR ICT[Text Word] OR wireless[Text Word]

CINAHL

Limits English, 2007
Hits 285
21/12/16

Qualitative

ethnolog* OR stories* OR story* OR content analys*OR ethnographic*OR observational methods OR participant observation OR field notes OR experiences OR narrative* OR discourse OR process evaluation OR service need* OR feelings OR ethnopsychology OR focus groups OR behavioral research OR behavioural research OR narration OR satisfaction OR dissatisfaction OR meanings OR meaning OR perspectives OR perspective OR perceived OR perceives OR perceive OR perceptions OR perception OR views OR view OR qualitative OR interviewed OR interviewing OR interviewer OR interviews OR interview OR comprehension OR opinions OR opinion OR expectations OR expectation OR thoughts OR narratives OR standpoint OR standpoints OR viewpoints OR viewpoint OR thematic analysis OR phenomenol* OR grounded theory OR grounded studies OR grounded research OR constant comparative OR constant comparison OR field study OR field studies OR field research OR biographical method OR theoretical sampl* OR open-ended OR open ended OR life world OR life-world OR conversation analysis OR conversational analyses OR theoretical saturation OR thematic analys* OR action research

Search Title, Abstract or Word in subject heading

Disease

Chronic OR long term OR persistent OR non-communicable OR respiratory OR asthma OR cystic fibrosis OR lung disease OR diabetes OR cancer OR heart disease OR cardiovascular disease OR pain OR muscular OR joint OR musculoskeletal OR kidney disease OR arthritis OR depression OR depressed OR anxiety OR anxious OR mental health OR schizophrenia OR fibromyalgia OR sickle cell OR disability OR disabled OR autism OR Asperger's OR adhd OR attention deficit hyperactivity disorder OR pulmonary OR cardiac OR ischemic OR renal OR neurological OR osteoarthritis OR osteoporosis OR ankylosing spondylitis OR psoriatic arthritis OR rheumatoid arthritis OR inflammatory arthritis OR lupus OR systemic lupus erythematosus OR juvenile idiopathic arthritis OR juvenile chronic arthritis OR back pain OR neck pain OR stress OR psychological

Search Title, Abstract or Word in subject heading

Young people

Young OR adolescent OR adolescence OR student OR students OR teenager OR teenage OR teenagers OR teen OR teens OR young adult OR young adults OR child OR children OR pediatric OR paediatric

Search Title, Abstract or Word in subject heading

mHealth

Search Title, Abstract or Word in subject heading

eHealth OR mHealth OR smartphone OR smartphones OR cell phone OR cell phones OR text messaging OR text messages OR digital technologies OR digital technology OR mobile OR mobiles OR wireless OR ICT

PsycInfo

Limit 2007
Hits: 401
21/12/16

Qual

ethnolog* OR stories* OR story* OR content analys* OR ethnographic* OR observational methods OR participant observation OR field notes OR experiences OR narrative* OR discourse OR process evaluation OR service need* OR feelings OR ethnopsychology OR focus groups OR behavioral research OR behavioural research OR narration OR satisfaction OR dissatisfaction OR meanings OR meaning OR perspectives OR perspective OR perceived OR perceives OR perceive OR perceptions OR perception OR views OR view OR qualitative OR interviewed OR interviewing OR interviewer OR interviews OR interview OR comprehension OR opinions OR opinion OR expectations OR expectation OR thoughts OR narratives OR standpoint OR standpoints OR viewpoints OR viewpoint OR thematic analysis OR phenomenol* OR grounded theory OR grounded studies OR grounded research OR constant comparative OR constant comparison OR field study OR field studies OR field research OR biographical method OR theoretical sampl* OR open-ended OR open ended OR life world OR life-world OR conversation analysis OR conversational analyses OR theoretical saturation OR thematic analys* OR action research

Search Title, Abstract or Heading word

Disease

Chronic OR long term OR persistent OR non-communicable OR respiratory OR asthma OR cystic fibrosis OR lung disease OR diabetes OR cancer OR heart disease OR cardiovascular disease OR pain OR muscular OR joint OR musculoskeletal OR kidney disease OR arthritis OR depression OR depressed OR anxiety OR anxious OR mental health OR schizophrenia OR fibromyalgia OR sickle cell OR disability OR disabled OR autism OR Asperger$ OR adhd OR attention deficit hyperactivity disorder OR pulmonary OR cardiac OR ischemic OR renal OR neurological OR osteoarthritis OR osteoporosis OR ankylosing spondylitis OR psoriatic arthritis OR rheumatoid arthritis OR inflammatory arthritis OR lupus OR systemic lupus

erythematosus OR juvenile idiopathic arthritis OR juvenile chronic arthritis OR back pain OR neck pain OR stress OR psychological

Search Title, Abstract or Heading word

Young people

Young OR adolescent OR adolescence OR student OR students OR teenager OR teenage OR teenagers OR teen OR teens OR young adult OR young adults OR child OR children OR pediatric OR paediatric

Search Title, Abstract or Heading word

mHealth

Search Title, Abstract or Heading word

eHealth OR mHealth OR smartphone OR smartphones OR cell phone OR cell phones OR text messaging OR text messages OR digital technologies OR digital technology OR mobile OR mobiles OR wireless OR ICT

Embase

Limit
2007
English
Article, article in press, conference paper
Hits 534

21/12/16

EMTREE

Qualitative

‘qualitative research’ OR ‘semi structured interview’ OR ‘thematic analysis’ OR ‘ethnographic research’ OR ‘field study’ ‘personal experience’ OR ‘experience’ OR ‘discourse analysis’ OR ‘behavioral research’ OR ‘satisfaction’ OR ‘meaningful’ OR ‘perception’ OR ‘interview’ OR ‘comprehension’ OR ‘phenomenology’ OR ‘grounded theory’ OR ‘constant comparative method’ OR ‘open-ended questionnaire’ OR ‘action research’

Key words
Title, abstract

‘ethnology*’ OR ‘stories*’ OR ‘story*’ OR ‘content analys*’ OR ‘ethnographic*’ OR ‘observational methods’ OR ‘participant observation’ OR ‘field notes’ OR ‘experiences’ OR ‘narrative*’ OR ‘discourse’ OR ‘process evaluation’ OR ‘service need*’ OR ‘feelings’ OR ‘ethnopsychology’ OR ‘focus groups’ OR ‘behavioral research’ OR ‘behavioural research’ OR ‘narration’ OR ‘satisfaction’ OR ‘dissatisfaction’ OR ‘meanings’ OR ‘meaning’ OR ‘perspectives’ OR ‘perspective’ OR ‘perceived’ OR ‘perceives’ OR ‘perceive’ OR ‘perceptions’ OR ‘perception’ OR ‘views’ OR ‘view’ OR ‘qualitative’ OR ‘interviewed’ OR ‘interviewing’ OR ‘interviewer’ OR ‘interviews’ OR ‘interview’ OR ‘comprehension’ OR ‘opinions’ OR ‘opinion’ OR ‘expectations’ OR ‘expectation’ OR ‘thoughts’ OR ‘narratives’ OR ‘standpoint’ OR ‘standpoints’ OR ‘viewpoints’ OR ‘viewpoint’ OR ‘thematic analysis’ OR ‘phenomenol*’ OR ‘grounded theory’ OR ‘grounded studies’ OR ‘grounded research’ OR ‘constant comparative’ OR ‘constant comparison’ OR ‘field study’ OR ‘field studies’ OR ‘field research’ OR ‘biographical method’ OR ‘theoretical sampl*’ OR ‘open-ended’ OR ‘open ended’ OR ‘life world’ OR ‘life-world’ OR ‘conversation analysis’ OR ‘conversational analyses’ OR ‘theoretical saturation’ OR ‘thematic analys*’ OR ‘action research’

Disease

Emtree
‘non communicable disease’ OR ‘chronic disease’ OR ‘chronic pain’ OR ‘chronic patient’ OR ‘respiratory tract disease’ OR ‘asthma’ OR ‘cystic fibrosis’ OR ‘lung disease’ OR ‘diabetes mellitus’ OR ‘neoplasm’ OR ‘heart disease’ OR ‘cardiovascular disease’ OR ‘pain’ OR ‘muscle’ OR ‘joint’ OR ‘musculoskeletal disease’ OR ‘kidney disease’ OR ‘arthritis’ OR ‘depression’ OR ‘anxiety’ OR ‘mental health’ OR ‘schizophrenia’ OR ‘fibromyalgia’ OR ‘sickle cell’ OR ‘disability’ OR ‘autism’ OR ‘attention deficit disorder’

Key words

‘Chronic’ OR ‘long term’ OR ‘persistent’ OR ‘non-communicable’ OR ‘respiratory’ OR ‘asthma’ OR ‘cystic fibrosis’ OR ‘lung disease’ OR ‘diabetes’ OR ‘cancer’ OR ‘heart disease’ OR ‘cardiovascular disease’ OR ‘pain’ OR ‘muscular’ OR ‘joint’ OR ‘musculoskeletal’ OR ‘kidney disease’ OR ‘arthritis’ OR ‘depression’ OR ‘depressed’ OR ‘anxiety’ OR ‘anxious’ OR ‘mental health’ OR ‘schizophrenia’ OR ‘fibromyalgia’ OR ‘sickle cell’ OR ‘disability’ OR ‘disabled’ OR ‘autism’ OR ‘Asperger’ OR ‘adhd’ OR ‘attention deficit hyperactivity disorder’ OR ‘pulmonary’ OR ‘cardiac’ OR ‘ischemic’ OR ‘renal’ OR ‘neurological’ OR ‘osteoarthritis’ OR ‘osteoporosis’ OR ‘ankylosing spondylitis’ OR ‘psoriatic arthritis’ OR ‘rheumatoid arthritis’ OR ‘inflammatory arthritis’ OR ‘lupus’ OR ‘systemic lupus erythematosus’ OR ‘juvenile idiopathic arthritis’ OR ‘juvenile chronic arthritis’ OR ‘back pain’ OR ‘neck pain’ OR ‘stress’ OR ‘psychological’

Young adult

Emtree
'Young adult' OR 'Adolescent' OR 'Juvenile' OR 'Student'

Keywords

'Young' OR 'adolescent' OR 'adolescence' OR 'student' OR 'students' OR 'teenager' OR 'teenage' OR 'teenagers' OR 'teen' OR 'teens' OR 'young adult' OR 'young adults' OR 'child' OR 'children' OR 'pediatric' OR 'paediatric'

mHealth

Emtree
'Telehealth' OR 'mobile application' OR 'smartphone' OR 'mobile phone' OR 'text messaging'

Keywords

'eHealth' OR 'mHealth' OR 'smartphone' OR 'smartphones' OR 'cell phone' OR 'cell phones' OR 'text messaging' OR 'text messages' OR 'digital technologies' OR 'digital technology' OR 'mobile' OR 'mobiles' OR 'wireless' OR 'ICT'

Scopus

Limits
2007
English
Article, conference paper

Hits
1935

Qual

"ethnolog*" OR "stories*" OR "story*" OR "content analys*" OR "ethnographic*" OR "observational methods" OR "participant observation" OR "field notes" OR "experiences" OR "narrative*" OR "discourse" OR "process evaluation" OR "service need*" OR "feelings" OR "ethnopsychology" OR "focus groups" OR "behavioral research" OR "behavioural research" OR "narration" OR "satisfaction" OR "dissatisfaction" OR "meanings" OR "meaning" OR "perspectives" OR "perspective" OR "perceived" OR "perceives" OR "perceive" OR "perceptions" OR "perception" OR "views" OR "view" OR "qualitative" OR "interviewed" OR "interviewing" OR "interviewer" OR "interviews" OR "interview" OR "comprehension" OR "opinions" OR "opinion" OR "expectations" OR "expectation" OR "thoughts" OR "narratives" OR "standpoint" OR "standpoints" OR "viewpoints" OR "viewpoint" OR "thematic analysis" OR "phenomenol*" OR "grounded theory" OR "grounded studies" OR

“grounded research” OR “constant comparative” OR “constant comparison” OR “field study” OR “field studies” OR “field research” OR “biographical method” OR “theoretical sampl*” OR “open-ended” OR “open ended” OR “life world” OR “life-world” OR “conversation analysis” OR “conversational analyses” OR “theoretical saturation” OR “thematic analys*” OR “action research”

Search Title, Abstract or Keyword

Disease

“Chronic” OR “long term” OR “persistent” OR “non-communicable” OR “respiratory” OR “asthma” OR “cystic fibrosis” OR “lung disease” OR “diabetes” OR “cancer” OR “heart disease” OR “cardiovascular disease” OR “pain” OR “muscular” OR “joint” OR “musculoskeletal” OR “kidney disease” OR “arthritis” OR “depression” OR “depressed” OR “anxiety” OR “anxious” OR “mental health” OR “schizophrenia” OR “fibromyalgia” OR “sickle cell” OR “disability” OR “disabled” OR “autism” OR “Aspergers” OR “adhd” OR “attention deficit hyperactivity disorder” OR “pulmonary” OR “cardiac” OR “ischemic” OR “renal” OR “neurological” OR “osteoarthritis” OR “osteoporosis” OR “ankylosing spondylitis” OR “psoriatic arthritis” OR “rheumatoid arthritis” OR “inflammatory arthritis” OR “lupus” OR “systemic lupus erythematosus” OR “juvenile idiopathic arthritis” OR “juvenile chronic arthritis” OR “back pain” OR “neck pain” OR “stress” OR “psychological”

Search Title, Abstract or Keyword

Young people

“Young” OR “adolescent” OR “adolescence” OR “student” OR “students” OR “teenager” OR “teenage” OR “teenagers” OR “teen” OR “teens” OR “young adult” OR “young adults” OR “child” OR “children” OR “pediatric” OR “paediatric”

Search Title, Abstract or Keyword

mHealth

Search Title, Abstract or Keyword

“eHealth” OR “mHealth” OR “smartphone” OR “smartphones” OR “cell phone” OR “cell phones” OR “text messaging” OR “text messages” OR “digital technologies” OR “digital technology” OR “mobile” OR “mobiles” OR “ICT” OR “wireless”

Grey literature

ProQuest
19/01/16
Limit
2007
English

454 hits

Qual

ethnolog* OR stories* OR story* OR content analys* OR ethnographic* OR observational methods OR participant observation OR field notes OR experiences OR narrative* OR discourse OR process evaluation OR service need* OR feelings OR ethnopsychology OR focus groups OR behavioral research OR behavioural research OR narration OR satisfaction OR dissatisfaction OR meanings OR meaning OR perspectives OR perspective OR perceived OR perceives OR perceive OR perceptions OR perception OR views OR view OR qualitative OR interviewed OR interviewing OR interviewer OR interviews OR interview OR comprehension OR opinions OR opinion OR expectations OR expectation OR thoughts OR narratives OR standpoint OR standpoints OR viewpoints OR viewpoint OR thematic analysis OR phenomenol* OR grounded theory OR grounded studies OR grounded research OR constant comparative OR constant comparison OR field study OR field studies OR field research OR biographical method OR theoretical sampl* OR open-ended OR open ended OR life world OR life-world OR conversation analysis OR conversational analyses OR theoretical saturation OR thematic analys* OR action research

Search Anywhere except full text

Disease

Chronic OR long term OR persistent OR non-communicable OR respiratory OR asthma OR cystic fibrosis OR lung disease OR diabetes OR cancer OR heart disease OR cardiovascular disease OR pain OR muscular OR joint OR musculoskeletal OR kidney disease OR arthritis OR depression OR depressed OR anxiety OR anxious OR mental health OR schizophrenia OR fibromyalgia OR sickle cell OR disability OR disabled OR autism OR Asperger$ OR adhd OR attention deficit hyperactivity disorder OR pulmonary OR cardiac OR ischemic OR renal OR neurological OR osteoarthritis OR osteoporosis OR ankylosing spondylitis OR psoriatic arthritis OR rheumatoid arthritis OR inflammatory arthritis OR lupus OR systemic lupus erythematosus OR juvenile idiopathic arthritis OR juvenile chronic arthritis OR back pain OR neck pain OR stress OR psychological

Search Anywhere except full text

Young people

Young OR adolescent OR adolescence OR student OR students OR teenager OR teenage OR teenagers OR teen OR teens OR young adult OR young adults OR child OR children OR pediatric OR paediatric

Search Anywhere except full text

mHealth

Search Anywhere except full text

eHealth OR mHealth OR smartphone OR smartphones OR cell phone OR cell phones OR text messaging OR text messages OR digital technologies OR digital technology OR mobile OR mobiles OR wireless OR ICT

Epistemonikus

Could not handle the long search string which had to be shortened

Limit 2007

747 hits

1 study was eligible, but had already been found elsewhere (Stinson 2013 Journal of medical Internet research)

Qualitative

(ethnology OR ethnologies OR ethnographic OR ethnographical OR experiences OR narrative OR narratives OR discourse OR feelings OR focus groups OR narration OR satisfaction OR dissatisfaction OR meanings OR meaning OR perspectives OR perspective OR perceived OR perceives OR perceive OR perceptions OR perception OR views OR view OR qualitative OR interviewed OR interviewing OR interviewer OR interviews OR interview OR thematic analysis OR phenomenology OR phenomenological OR grounded theory OR grounded studies OR grounded research OR theoretical saturation OR thematic analysis OR thematic analyses OR action research) AND

Disease

(Chronic OR long term OR persistent OR non-communicable OR respiratory OR asthma OR cystic fibrosis OR lung disease OR diabetes OR cancer OR heart disease OR cardiovascular disease OR pain OR muscular OR joint OR musculoskeletal OR kidney disease OR arthritis OR depression OR depressed OR anxiety OR anxious OR mental health OR schizophrenia OR fibromyalgia OR sickle cell OR disability OR disabled OR autism OR Asperger OR adhd OR arthritis OR back pain OR neck pain OR stress OR psychological) AND

Young adults
(Young OR adolescent OR adolescence OR student OR students OR teenager OR teenage OR teenagers OR teen OR teens OR young adult OR young adults OR child OR children OR pediatric OR paediatric)

mHealth

AND (eHealth OR mHealth OR smartphone OR smartphones OR cell phone OR cell phones OR text messaging OR text messages OR digital technologies OR digital technology OR mobile OR mobiles OR wireless OR ICT)

KT Strategies Database

Database included 196 entries, hand searched 3/2/17, no hits obtained
