# Supplementary material for: End User and Implementer Experiences of mHealth Technologies for Noncommunicable Chronic Disease Management in Young Adults: Systematic Review
Source: J Med Internet Res. 2017 Dec 12;19(12):e406. doi: 10.2196/jmir.8888 (PMC5743925; doi:10.2196/jmir.8888)
Supplement: Multimedia Appendix 4 [file jmir_v19i12e406_app4.pdf]

Supplemental File 4

Themed categories for end-users' experiences of mHealth technologies

1. *Experiences of mHealth technologies functionality*

(a) mHealth functionality to support self-management

The functionality of the mHealth technologies supported self-management of a range of NCDs. These technologies enabled young people to improve their understanding of their condition, to monitor and track symptoms; to access early intervention and professional advice in a timely fashion in response to disease status; to support treatment adherence; to facilitate self-awareness/self-reflection of disease status; and to provide relevant information, support and reassurance about planning for emergencies and flare ups/exacerbation.

**Finding**	**Illustration**
Using a combination of self-management features is what seemed to have the strongest relationship with increasing self-observation and self-judgment among adolescents.<br><br>Unequivocal	I used the symptoms, triggers, and notes, cause—because with the symptoms, it can—it pretty much tells how—like what I'm feeling at that time like throughout the day and the triggers is like if I have a flare up or, uh, an attack or—then it'll—it'll help, it'll show like what—what caused it in the notes because it just—I can just put down everything that happened throughout the whole day. **Carpenter 2016** *Page 515, column 2**
Participants also reported using the peak flow graphics to better self-judge their asthma.<br><br>Unequivocal	"Like it—it really did help me out, um, and to know about the progress of my-of my asthma... it let me like know more of how my asthma was going during the weeks and—and days." **Carpenter 2016** *Page 513 column 2**
Adolescents linked the trigger feature to increased self-observation, in that they could track their exposure to triggers over time, which led them to engage in better environmental control behaviors.<br><br>Unequivocal	"The triggers, um, I thought it was good because it would help you keep track of like what triggered it before, so you would know to stay away from it or stay indoors if it's like a certain type of plant blooming or something. And it would help you, uh, remember that for the future years, so you could, um, remember to stay away from it." **Carpenter 2016** *Page 514 column 1**

The convenience of being able to document information electronically and track progress over time were cited as benefits of this feature.<br><br>Unequivocal	"because every time I enter it at the device, I wanted to go back to see how it was, all I have to do is go to the diary and see how it's been over a time period." **Carpenter 2016** *Page 514 column 1**
Adolescents appreciated being able to visualize how their asthma was changing over time.<br><br>Unequivocal	I liked the chart. It kind of helped me see what I was, uh, how I've been doing over the course of time. Um, I normally didn't really monitor it that much, I kind of let my mom did it, and this way if I were using this, I could kind of tell myself how I was doing on my own. **Carpenter 2016** *Page 514 column 1*
The charting feature seemed to increase both self-observation and self-judgment, as adolescents liked to see if their asthma was getting better or worse.<br><br>Unequivocal	"The chart, cause I can like sc-, I can watch it, I can scale my asthma and I can see if it's worse or if it's getting better, or if it's really serious I need to do something about it, it helps me. Um-hum." **Carpenter 2016** *Page 514 column 1**
This feature [self-check] was helpful for increasing adolescents' ability to self-judge their asthma severity<br><br>Unequivocal	"I think it's really good because you get to take this test over and over again, and like over time, maybe your score might get, your score might get higher and your score might get higher and that might be a good thing. And you'd be really – and like I like the test because you can really see if your, your asthma is getting better." **Carpenter 2016** *Page 514 column 2*
Record of symptoms to see change and symptom patterns<br><br>Unequivocal	''Can see how symptoms change for other cycles.'' **Gibson 2010** *Page 349*, *table 3*
Increased awareness of symptoms and triggers<br><br>Unequivocal	"You could like share like [...] your symptoms with the machine or whatever and it helped you realize what your symptoms were – actually were. It's [...] sometimes you don't really um [...] like [...] realize that you've had symptoms – you actually have to think about it." **Rhee 2014** *Page 67*, *column 2*

Improved asthma self-management and medication adherence<br><br>Unequivocal	“I feel like it could help you manage your asthma because it like tells you when like you’re having flare-ups and what time of day and then that can help you find out why because you can reflect and think back what you were doing then or what you were exposed to.” **Rhee 2014** *Page 68*, *Column 1**
Adolescents also discussed how they were able to take action to keep their asthma under control when the self-check feature result indicated that they were having problems.<br><br>Unequivocal	"I think what this is, this is very good. It helps you, um, track it so maybe you can catch your – catch it before it gets bad, you know, saying, you know, my – I wrote down I have a lot of symptoms all this week. Maybe, maybe I should, you know, check and see if Ineed to start doing my Xopenex and, you know, maybe do more upkeep I guess." **Carpenter 2016** *Page 514 column 2*
Reminders helped adolescents with forgetfulness and helped them become more observant of their medication-taking behaviors.<br><br>Unequivocal	"And I always remember to take my medicine easier with this app so I think that will help out. Because if I could continue to take my medication on sort of, uh, a consistent flow it makes it easier. And so overtime, I think it will help me control my asthma." **Carpenter 2016** *Page 513 column 2**
Adolescents liked having all the information about what to do in case of an emergency in one location that was easy to share with others, so they could get the support they need in case of an asthma attack. In this way, the plan was linked to both technical advice and social support.<br><br>Unequivocal	"You know, you can just pull it up and say, you know, I have all the information and I have it with me. And maybe in the case of a flare, flare, really your friend can see it and say, oh, it says her emergency plan is to do this, this, and this, so I can help her do this, this, and this." **Carpenter 2016** *Page 514 column 2*
To see and reflect using pictures improves understanding and knowledge and affects self-treatment.<br><br>Credible	The most important factor for coping was seen as control of improved daily glucose values. DiaMob was reported as giving them better insight into what causes changes in glucose measurements. They reported better coping and not least increased motivation to succeed. **Froisland 2015** *Page 545*, *Table 1*
Improved comprehension and increased feeling of managing the self-treatment.<br><br>Credible	Told that they understood diabetes theory before start of the study. However, reported that the picture app changed their understanding of cornerstones of treatment. This was reported as giving them an increased feeling of being able to manage the disease. **Froisland 2015** *Page 545*, *Table 1*
Support and reassurance for young people/families from being monitored.	‘‘It would just sort of reassure them a lot of the time that what they are feeling is normal, and then if there

Unequivocal	is anything out of ordinary then someone's gonna pick up on it quite quickly, and probably for their parents, it would put their minds at rest as well.'' **Gibson 2010** *Page 349*, *table 3*
It provided a safety net that gave them a sense of protection because it made it easy for them to access their physician with questions and concerns.<br><br>Unequivocal	"The fact that you have someone to support you—someone who knows the subject, and if you get into difficulties you can get an answer—it gives a certain feeling of security." **Froisland 2012** *ePub*
Early intervention<br><br>Unequivocal	''You can nip symptoms in the bud.'' **Gibson 2010** *Page 349*, *table 3*
Accessibility of asthma-related advice<br><br>Unequivocal	"I think it would help them just 'cause if they needed someone's opinion or they didn't know what to do they can text you and they'd send back a quick answer." **Rhee 2014** *Page 68*, *Column 2*

(b) *mHealth functionality to support person-centred clinical encounters.*

The functionality of the mHealth technologies supported a person-centred clinical encounter by enabling accurate provision of clinically-relevant information to treating practitioners (portability, and accuracy of data over a cumulative period of time), and helped to set the focus the clinical encounter.

**Finding**	**Illustration**
Perceived Usefulness of App for Preparing for or setting the Focus of Clinic Appointment<br><br>Credible	Over half of reviewers [...] thought their chosen app was useful or very useful for preparing for or setting the focus of their clinic appointment. **Ashurst 2014** *ePub*
Improve health professional's knowledge and understanding<br><br>Unequivocal	''They'll [doctors and nurses] be able to know exactly what is happening.'' **Gibson 2010** *Page 349*, *table 3**
Aid communication in consultations with professionals<br><br>Unequivocal	''It can help them remember their symptoms because when you come in for the first day of a cycle, doctors always ask you about the symptoms over like the last cycle, and it's sometimes kind of hard to remember what you had on each cycle. So if it's like stored in the thingy, then you can tell them a little bit.'' **Gibson 2010** *Page 349*, *table 3*

Adolescents liked that they could share the [school] form with their school nurse so he or she could know what medications they were taking. Like the emergency plan, the school form seemed most linked to social support and technical advice.<br><br>Unequivocal	"I could give it to my school if there's a problem with my asthma, they can say, 'Oh, well she did send us this document saying that she has asthma, so we need to let her take her medicine,' so that's a good thing." **Carpenter 2016** *Page 515 column 1**
Teens thought the [Doctor] report "travels a lot better" than paper and is a nice way to summarize their asthma experience in a concise format to give to their providers<br><br>Unequivocal	"I think that was good…so like if your doctor just wonders how you're doing when he doesn't see you, you could, you could send him the chart and he could see how you've been doing." **Carpenter 2016** *Page 515 column 1**

2. Acceptance of mHealth technologies
(a) Perceptions on technical usability (how it's working now and how they perceive optimisation)

Users identified technical aspects of the mHealth technologies that affected usability and made suggestions for optimisation/improvement as it relates to scalability and implementation.

**Finding**	**Illustration**
Although the participants described the applications as highly usable in general, they also identified particular problems that required resolution.<br><br>Unequivocal	"The Diamob app didn't work at the end of the project. The glucometer with Bluetooth worked, but batteries ran out of power quickly." **Froisland 2012** *ePub**
[…] several participants indicated that the Web-based SMS system was unduly cumbersome.<br><br>Unequivocal	"But what is cumbersome is that you have to access that Internet browser on the mobile. I would prefer to send normal SMS on the phone…that would make it even easier if you could access it using the usual SMS [on the phone]." **Froisland 2012** *ePub**
All the adolescent participants used the verb "to see" in relation to the first application's functionality.<br>Unequivocal	"I think it is a lot easier to understand and to have it explained when I can see things." **Froisland 2012**
Intentions to Use Again and Recommend to a Friend<br><br>Credible	Overall, reviewers indicated that the apps were worth trialling but a few felt improvements or amendments were needed before regular use. **Ashurst 2014** *ePub**

Useful App Features<br><br>Credible	By app function, the most useful features reported in qualitative feedback were: for data logging apps (1) setting targets and viewing trends, (2) ease of recording and tracking data, and (3) data storage in one mobile location without need for logbook or pen/ paper; for the insulin dose calculation apps (1) simplicity and ease of use, (2) accuracy and trust of calculator, and (3) all in one calculation (carbs and insulin); for the notes/ agenda setting apps (1) the topic prompts to identity and remember what to discuss at appointment, (2) simple layout and ease of use, and (3) ability to document and review notes. **Ashurst 2014** *ePub**
The participants agreed that the applications were highly usable, but requested the possibility of downloading the applications to their own mobile phones in the future.<br><br>Credible	With 1 exception, the participants indicated that if the applications were available for downloading to their own phones, they would continue to use the applications. **Froisland 2012** *ePub*

(b). *Perceptions and experiences around acceptability and feasibility*

Users identified characteristics of mHealth technologies that aligned with their preferences for management support, specifically apps that were intuitive (self-explanatory and simple to understand) and practical self-management information that was immediately usable. While barriers were identified, the use of mHealth technologies was still feasible as end-users were able to adjust to the use of mHealth technologies with their daily routines.

**Finding**	**Illustration**
Although adolescents thought electronically documenting information was convenient, other barriers could limit use of certain features, particularly the peak flow feature.<br><br>Unequivocal	I think most people just don't want to do them [peak flows]. And you don't want to have to – because first, you have to, you know, use it. You have to use it three times and you really start coughing, hacking after you've used it. Most people don't like peak flows. And then in addition to actually having to do the peak flow, you – if you want to see how you're doing really, you have to document it. **Carpenter 2016** *Page 515, column 1**
mASMAA feasibility and acceptability<br><br>Unequivocal	Adolescents were able and willing to make adjustment to their routines to accommodate mASMAA and became accustomed to interactions with mASMAA easily ("You get used to it and it becomes routine"; "I feel like it becomes normal, just like … an instinct to do it") **Rhee 2014** *Page 67, column 2**

All patients who were questioned reported that they would recommend the application to others<br><br>Unequivocal	"… because it really helps to see how you've been doing too actually, not just like, you know, go on day by day. But it does help you see and reflect on how you're doing. And if you did something the other day that helped, will help you make you feel better and so on." **Bagott 2012** *Table 5*
Patients' preference for the use of the VAS or face scales to rate pain and nausea were fairly evenly divided.<br><br>Credible	Those who preferred the VAS appreciated the opportunity to select a more precise level of pain or nausea, compared to only a few options on the faces scales. Those who preferred the faces scales commented that the diagrams depicted how they were feeling. **Bagott 2012** *ePub*
Ease of Use Per App<br><br>Credible	Reviewers' felt the easiest to use apps were self-explanatory and simple to understand. The other apps were also considered easy to use but with some suggestions to improve the user-interface. **Ashurst 2014** *ePub**
[...] stated that they appreciated simple and practical self-management advice more than large amounts of information that was not relevant to their immediate situation.<br><br>Unequivocal	"It is more about those messages and the information. It has been practical advice, easy to understand, simple facts that are very nice to know. It is better to have it in such small portions instead of reading a lot of information, then everything is poorly read and poorly understood. I liked the way the information was given." **Froisland 2012** *ePub**

3. The importance of co-design

End-users identified the critical importance of co-design of mHealth technologies. Both including (a) intrapersonal and (b) extrapersonal factors were highlighted as considerations for tailoring design.

Finding	Illustration
However, intrapersonal factors, such as inadequate<br>knowledge of one's triggers, limited some adolescents' use of the [triggger and symptom tracking] feature<br><br>Unequivocal	"I really don't know what my triggers are, so I really didn't use it that much." **Carpenter 2016** *Page 514 column 1**
[...] adolescents who felt like they were already doing a good job managing their asthma were less likely to spend time using the apps since they did not have symptoms or triggers they wanted to document.<br><br>Unequivocal	"Because, like my asthma is well-controlled, so like a lot of the stuff here I don't really need, but maybe like other people who have it worse will like probably need it more." **Carpenter 2016** *Page 515, column 1**

[…] competing demands limited adolescents' ability to use the apps […]<br><br>Credible	[…] one participant noted that she was too busy to use an asthma app. **Carpenter 2016** *Page 515*, *column 1**
The Importance of the Apps Being Created by YPD<br><br>Credible	[…] much importance was placed on app design (not necessarily development) by diabetic peers because of a mutual understanding of the needs, condition and experiences in order for the apps to offer the most accurate features and details. **Ashurst 2014***
Social settings change action readiness with regard to acceptance of disease and self-treatment. DiaMob increased social acceptance<br><br>Credible	Most adolescents in the study felt in charge of their own life, however they talked about acceptance as an important factor. Acceptance of own disease and treatment and also acceptance from important others like friends to treatment while in different social settings. **Froisland 2015** *Page 545*, *Table 1**

4. Perceptions of benefit
(a) *self-efficacy (locus of control)*

End-users identified that mHealth technologies positively influenced self-efficacy.

Finding	Illustration
Improved sense of control over asthma and its management<br><br>Credible	"[…] adolescents reported increased independence during the trial, as indicated in their improved self-management (eg, taking medications) without parents' prompting." **Rhee 2014** *Page 68*, *Column 2**
Increased control for young people<br><br>Unequivocal	''I felt in control and I liked that you could see if your temperature had improved.'' **Gibson 2010** *Page 349*, *table 3**
SMS solutions lower the threshold for contact and give adolescents a sense of "being in charge."<br><br>Credible	The direct contact with those they trust was reported as important. To know that they got an answer back, gave a feeling of acceptance and to be paid attention to. **Froisland 2015** *Page 545*, *Table 1**

(b) *Empowerment*

mHealth technologies were perceived as empowering condition management by young people

Finding	Illustration

Positive feedback leads to mastery and increases empowerment. Negative feedback leads to opposition.<br><br>Credible	Positive response from people who know the disease is important to feel empowered. The SMS application increased the possibility for response directly from their health care professional. **Froisland 2015** *Page 545*, *Table 1**
The participants also reported feeling empowered in that they could access the health care practitioner so readily<br><br>Unequivocal	"It has been pretty good to know that if I have an issue, then I can just send a message...Instead of calling Mom or Dad and ask them to call [the physician], and when they have the answer it might be an answer to something I was not wondering about." **Froisland 2012** *Page 513 column 2**
Patients are empowered and can change their own lives when they are able to integrate knowledge and resources to take rational decisions. Through experience they are further able to evaluate the effectiveness of their decisions.<br><br>Credible	DiaMob gave them a visual and tangible understanding of how physical activity, food intake, and insulin dosage, interact and affect postprandial glucose measurements. This gave them a new start to change the direction of their own treatment. SMS solution gave them a feeling of being in charge and closer to the health care practitioners. **Froisland 2015** *Page 545*, *Table 1*
Using the app also resulted in several positive self-reactions for adolescents, including feeling more confident that they could take more responsibility for managing their asthma, obtaining the support they need to manage asthma, and communicating more effectively with others about their asthma.<br><br>Unequivocal	"It kind of keeps me to where I can see what I've done, instead of it just being in my mom or my doctor knowing how far I've come, where – if I'm getting better or worse, if I'm normal for myself or anything, I can kind of keep myself in check." **Carpenter 2016** *Page 516*, *column 1*

* indicates supporting quotes also included in full text paper
mASMAA= Mobile phone-based asthma self-management aid for adolescents; VAS=visual analogue scale; SMS=short message service; YPD=young people with diabetes
