# Supplementary material for: End User and Implementer Experiences of mHealth Technologies for Noncommunicable Chronic Disease Management in Young Adults: Systematic Review
Source: J Med Internet Res. 2017 Dec 12;19(12):e406. doi: 10.2196/jmir.8888 (PMC5743925; doi:10.2196/jmir.8888)
Supplement: Multimedia Appendix 5 [file jmir_v19i12e406_app5.pdf]

Supplemental File 5

Themed categories showing implementers' perceptions and experiences of mHealth technologies

1. mHealth technology characteristics that support young people's management of NCDs

(a) functional aspects of design that support end-users' management

Implementers identified a range of design features that were perceived to support end-users (young people's) management of their conditions. This included tracking side effects and symptoms, accessing information, motivation, alerts for deteriorating health condition, knowledge and self-awareness, habituation of components of self-management (e.g.; medication management), and enabling bilateral communication, and as overcoming communication deficiencies.

**Finding**	**Illustration**
Symptom Tracking to Improve the Quality of Clinic Appointments<br><br>Unequivocal	"This way you can look back over the previous 4 weeks or 3 months and focus on questions such as—"you scored sleep a 2 here, what was happening at the time that made it so unsettled?" It should help parents to be more productive in giving the information we need." **Simons 2016** *Page 9*, *column 1*
Diary and chart features<br><br>Credible	Most parents and clinicians believed the diary and chart were crucial features for management of the child's asthma. **Geryk 2016** *ePub*
Monitoring and Tracking Side Effects and Symptoms<br><br>Unequivocal	Graphs would be useful for example for… patients who stop taking meds but parents and teachers say they have improved. It might help to have the parent and teacher graphs to see. **Simons 2016** *Page 10*, *column 2*
Supporting Greater Self-Management<br><br>Credible	"Participants saw the potential for RMT to provide the ability to easily monitor symptoms, chart them over time, and identify any patterns or unusual behaviors. This would increase people's knowledge, self-awareness,

	and understanding of and confidence in dealing with their condition." **Simons 2016** *Page 9, column 1*
Medication Reminder Feature<br><br>Credible	Most clinicians felt that the medication reminder feature would help their patients a lot, particularly with their controller medications, as they saw medication nonadherence as an important clinical issue. **Geryk 2016** *ePub*
Organization Aid<br><br>Unequivocal	The difficulties I come across, [are that] young people are on medication and they tend to run out at the end of the month and their behavior will go sky high, and it will take them a week to get all the medication back into their system. I think it would be really useful if somewhere in the app, say when they're…near the end [they receive a message saying] "You need to put in a request for repeat prescription." [HCP, Site 3] **Simons 2016** *Page 10, column 1*
Time was an important factor in interaction<br><br>Unequivocal	"You can send text messages at a time that suits you and at the pace that you need. That is a very positive thing" **Buchholz 2013** *Page 93, column 2*
Physicians believe that mobile technology could improve communication by reaching adolescents directly.<br><br>Unequivocal	'' .. . teenagers are busy and communication is limited and I think using technology will improve communication. They'll listen more. I mean, I think they read their texts, you know, and I think reading a short text is much more beneficial and reminder systems on an everyday, I mean, doing something the same way for 2 weeks makes it a habit.'' **Schneider 2016** *Page 156*

School Form Feature<br><br>Credible	Some parents and clinicians felt that communicating asthma care between various entities using the school form feature would be very convenient. **Geryk 2016** *ePub*
The use of mobile technology by physicians and patients turns this platform into a feasible, accessible, and acceptable outreach mode.<br><br>Unequivocal	‘‘Teens probably most of them it would have to be some type of an e-mail or text messaging ‘cause I feel like most of our teen patients have access to that type of information. That, I think, is definitely a patient population who may be more willing to respond to you.’’ **Schneider 2016** *Page 157*
Physicians believe that repeated reminders assist adolescents in practicing and routinizing their asthma action plan and developing self-management skills.<br><br>Unequivocal	‘‘Y’know, to use the Aero Chamber with it, y ’know, or something. But I think the best reminders will be, maybe, during cold season or something, to remember—just a reminder that said, y ‘know, to use your—‘‘These are the symptoms. Remember to use your Albuterol.’’ So, I think just reminders are going to be best for, maybe, just a little blurb related to the symptoms and for follow-up appointments. It might be for acute follow-up visits or it might be for routine visits.’’ **Schneider 2016** *Page 158*
Reliable, Trustworthy, and Tailored Information<br><br>Unequivocal	I’m feeling dizzy—[the] advice would be to go to see your GP”. **Simons 2016** *Page 10, column 1*

The possibility to use remote communication affected the users' feeling of safety in daily life.<br><br>Unequivocal	“She has found the courage to do things she didn’t dare before …for example she was scared to be home alone” **Buchholz 2013** *Page 93, column 1*
A Coach/Supporter/Motivator<br><br>Unequivocal	[It] could have some information based on how long the process should take with messages such as “you may not be seeing any improvements yet, but stick with it”… or you could have messages to parents, such as “Derek might be struggling this week” **Simons 2016** *Page 10, column 1*
The speech synthesis was indispensible to users when listening to an incoming message.<br><br>Unequivocal	“When she receives them, that’s what has been the single most important thing for her in the phone” **Buchholz 2013** *Page 92, column 1*
Speech synthesis was important for the user in the construction of a message.<br><br>Unequivocal	“He has great help from the synthetic speech and he is markedly disturbed when it doesn’t really sound like he wants it to” **Buchholz 2013** *Page 92, column 1*
Physicians believe that alerts regarding a worsening asthma condition and the need for follow-up are crucial for reducing the number and severity of exacerbations.<br><br>Unequivocal	‘‘I would probably have—probably tie the—maybe a color scheme. If it comes over and the numbers are in the red then a response comes back in the red, too, that’s basically saying, y ‘know, instructions. ‘‘You need to do this and this and—’’ kind of like a little triage form. ‘‘Do this and this. If not this, then you need to report to the ER.’’ **Schneider 2016** *Page 158*

(b) *How technical characteristics of mHealth can help their delivery of clinical care*

Implementers identified several technical features that they believed would assist their delivery of clinical care and optimise their engagement with end-users through communication, reminders and focussing clinical encounters

Finding	Illustration
Communication /Information Sharing*<br><br>Credible	Clinicians felt that use of the app could lead to a better medical appointment both in terms of efficiency, patient-centered care, and decision making. Multiple clinicians expressed data security concerns (eg, insecure email) or differed in their preference for information delivery method […] **Geryk 2016** *ePub*
They perceived text messaging and e-mails as efficient and preferred communication channels to connect with patients […]<br><br>Unequivocal	''I am a big fan of texting. I would love to just get everything texted. If I could just text data to people all day I would save hours of time.'' **Schneider 2016** *Page 156*
Doctor Appointment Reminder Feature<br><br>Unequivocal	Multiple clinicians mentioned that appointment noncompliance is a problem, one stating that "[a]ny extra reminder that families have that they have an appointment I think is helpful." **Geryk 2016** *ePub*

(c) *how mHealth technology can support positive behavioural change*

Implementers perceived mHealth technologies to positively influenced end-users to independently manage their condition and facilitate positive health behaviour change

Finding	Illustration
Monitoring and Supervision<br><br>Credible	Clinicians generally had positive things to say about the apps as a self-management tool to help parents and adolescents including the following: "hands-on" and provides a "more interactive or fun way to check on their asthma." **Geryk 2016** *ePub*
Physicians perceive positive feedback as essential for reinforcing behavior change and encouraging asthma control.<br><br>Unequivocal	''I mean if everything is going well, you could give them sort of positive feedback just like: ''Hey, keep up the great work.'' If not, you could be like: ''Are you taking your controller?'' **Schneider 2016** *Page 158*

The users' possibilities to manage things on their own increased with the use of TMSS<br><br>Unequivocal	“It gives a direct contact that doesn’t go through someone else and that in itself makes it a more independent way of communication” **Buchholz 2013** *Page 93*, *column 1*

2. Implementation challenges

(a) *Technical features as barriers to implementation at the clinical (micro) level*.

These included, accuracy of monitoring and limitation of task-specific capability.

Finding	Illustration
The device did not always live up to the users’ expectations about how it should compensate for his or her communication difficulties.<br><br>Unequivocal	“Basically he seems to think it’s good but he’s frustrated because he thinks …he has very high expectations and to this point he doesn’t feel they have been met” **Buchholz 2013** *Page 91*, *column 1**
Asthma Trigger Features<br><br>Unequivocal	this feature was more often criticized by parents and clinicians because of its lack of long-term monitoring and feedback capabilities. One clinician expressed the opinions of other participants when stating, “I don’t know what you’d [do] with it. Other than just be aware of it.” **Geryk 2016** *ePub**
The speech synthesis was sometimes a subject of irritation.<br><br>Unequivocal	“When we send texts to [him] we have to be careful with commas exclamation marks and question marks so that the synthetic speech will be clear” **Buchholz 2013** *Page 91*, *column 2*
Peak Flow Feature<br><br>Credible	Clinicians expressed some reservations to patient peak flow use, including worrying about the adolescent obtaining an accurate peak flow reading. **Geryk 2016** *ePub*

(b) *Organisational level (meso) barriers to implementation*.

These included internal regulations, resource allocation (remuneration and funding), integration into workflow, organisational climate and readiness for change, interoperability with existing IT systems

Finding	Illustration
Physicians also raised reimbursement of services outside their routine responsibilities as a barrier.<br><br>Unequivocal	‘‘The biggest thing is … a time issue, lack of reimbursement … for adding additional duties.’’ **Schneider 2016** *Page 157**
Right intervention; wrong setting<br><br>Unequivocal	“We see young people with severe mental health problems, including suicidal ideation, and I’m not sure it’s ideal for this group… Most self-harm is dealt with by family support workers and schools, and they are always looking for additional resources and tools to help with it.” **Owens 2016** *Page 7, column 1**
Access to Technology<br><br>Credible	“This included access to mobile devices, especially ones that would support any new app/software (all groups); […]; and capability for interoperability with NHS hardware.” **Simons 2016** *Page 9, column 2*
Perceived burdensomeness and technophobia<br><br>Unequivocal	“The general perception within the team is that using TeenTEXT is too much of an extra burden on top of our existing workload.” **Owens 2016** *Page 6, column 2**
Perceived Challenges of Incorporating RMT Into Clinical Care<br><br>Unequivocal	HCPs were keen on receiving digital information that coincided with patients’ appointments, but noted that “if you start getting notifications about patients from another area clinic it will take up too much time” **Simons 2016** *Page 9, column 2*
Organisational gatekeeping<br><br>Unequivocal	“The organisation doesn’t give clinicians any leeway. We need permission to try anything new and there are so many hoops to jump through before that happens.” **Owens 2016***
Context: CAMHS in crisis<br><br>Unequivocal	“CAMHS is overwhelmed at the moment… It may have been the wrong time to try something new…There have been so many organisational changes. Managers have left, there’s been the introduction of Child IAPT1 services and there are high rates of sickness absence. Tis does affect our ability to get involved with new projects.” **Owens 2016** *Page 5, column 2*

(c) *System level (macro) barriers to implementation.*

These related to regulatory frameworks and health information security

Finding	Illustration
Communication /Information Sharing*<br><br>Credible	Clinicians felt that use of the app could lead to a better medical appointment both in terms of efficiency, patient-centered care, and decision making. Multiple clinicians expressed data security concerns (eg, insecure email) or differed in their preference for information delivery method [...] **Geryk 2016** *ePub**
Sharing medical information electronically or by the Internet potentially creates security risks.<br><br>Unequivocal	‘Oh, I would love to do it by electronic means. The problem is that then you run into all the HIPAA problems.’’ **Schneider 2016** *Page 157**
Doctor Report Feature<br><br>Credible	However, clinicians mentioned specific communication-related concerns including the Health Insurance Portability and Accountability Act (HIPAA) and security concerns, not wanting to be handed the report by families in clinic, or seeing the feature as impractical with difficulties getting it to interface well with office technology and to link to a patient chart. **Geryk 2016** *ePub*

3. Adoption of mHealth technologies in a specific young population

a) Implementers perceived the need for training for end-users for some conditions and settings to facilitate adoption. For features to encourage adoption included using existing technology and hardware, and physical ease of handling devices.

Finding	Illustration
Overall, the design of the devices seemed to be satisfactory, since it was easy for the users to hold, see and point at the screen.<br><br>Unequivocal	“It has been easy to handle for him ...it has been easy also in terms of making adaptations (for the helper)” **Buchholz 2013** *Page 91, column 2**

TMSS was useful to be able to use mobile phone technology in daily life activities.<br><br>Unequivocal	"This is an aid that would be of help for a lot of people. I have many colleagues with clients who would need something similar maybe particularly adolescents that are becoming adults" **Buchholz 2013** *Page 92*, *column 2 **
The possibility to be able to use the same technology as others in society was important. By succeeding in using the devices, the users had the opportunity to express their views independently.<br><br>Unequivocal	"You might get a stronger self when you get to be a little more independent mainly with the texting but also using the calendar or the price calculator for keeping track of things" **Buchholz 2013** *Page 92*, *column 2*
It was important to blend in; to have a device that didn't look different from the every-man's.<br><br>Unequivocal	"Exciting a little more up to date ...modern ...or she would never have accepted it" **Buchholz 2013** *Page 91*, *column 1**
There was wish for the software to be installed in the users' regular phones.<br><br>Unequivocal	"Yes because if this software was installed in the regular phone I think she would use it more" **Buchholz 2013** *Page 91*, *column 1**

b) Features to encourage adoption included using existing technology and hardware, and ensuring design that supported ease of handling devices

Finding	Illustration
It was sometimes hard to know if and to what extent the users understood the purpose of the device before having gained experience of use.<br><br>Credible	"Yes, it was very abstract I think so when we could show him something more concrete he grasped it better" **Buchholz 2013** *Page 91*, *column 2**
The training needed to be a part of daily activities and actual communication situations.<br><br>Unequivocal	"It's important to find a situation where you really see the need of being able to text or a person you need to contact where a regular phone call won't work" **Buchholz 2013** *Page 92*, *column 1**

4. Co-design and tailoring

(a) importance of co-design: implementers identified the importance of working collaboratively with end-users to optimise functionality.

Finding	Illustration
The pre-designed phrases were valuable for initial training where the phrases served as suggestions for messages that could be sent.<br><br>Unequivocal	“She has great use of them and we have built upon her interests so she can easily reply to a text and she can also send a pre-designed text” **Buchholz 2013** *Page 93, column 2**
My Allergies and Emergency Plan Feature<br><br>Credible	One clinician brought up the benefits of using the feature for “engaging with them [patients]” including jointly inputting information into the plan and/or discussing what patients have previously input to ensure they are getting the correct guidance, especially regarding emergency situations. **Geryk 2016** *ePub**
Engagement in principle<br><br>Unequivocal	“I like the fact that the messages are written by them, so they’re supporting themselves… This fits with what we currently do, which is try and give them a sense of control.” **Owens 2016** *Page 5, column 1**
Self-Check Quiz Feature<br><br>Unequivocal	“[k]ids are involved. They are more willing to be self-advocates for themselves.” **Geryk 2016** *ePub*

(b) Implementers identified the need for the design of mHealth technologies to be adaptable to end-users, providing for tailored content and function.

Finding	Illustration
[…] with time, the devices were also used for face to face communication.<br><br>Unequivocal	“Yeah it’s like that. He has started to use it more for face to face communication. . . not just the text-messaging function but more as a communication device” **Buchholz 2013** *Page 94, column 1**

Physicians believe that tailoring messages responsive to preferences about delivery mode, time, and content would increase youth's receptiveness to the intervention<br><br>Unequivocal	''Don't forget to pretreat before you go out for soccer practice, or football practice,'' specific for that patient's sport I think would be even more, you know, something that's specific for that patient.'' **Schneider 2016** *Page 158**
Education<br><br>Unequivocal	"I think most of them engage in devices like this for entertainment, right? And so you want to have something that provides them an educational opportunity, um, but also something that they – they won't get bored with." **Geryk 2016** *ePub*

* indicates supporting quotes also included in full text paper
TMSS=text messaging with picture symbols and speech synthesis; NHS=National Health Service
